# Vascular endothelial growth factor signaling in health and disease: from molecular mechanisms to therapeutic perspectives

**DOI:** 10.1038/s41392-025-02249-0

**Published:** 2025-05-19

**Authors:** Chunsik Lee, Myung-Jin Kim, Anil Kumar, Han-Woong Lee, Yunlong Yang, Yonghwan Kim

**Affiliations:** 1 Department of R&D, GEMCRO Inc, Seoul, Republic of Korea; 2 Department of Biological Sciences and Research Institute of Women’s Health, Sookmyung Women’s University, Seoul, Republic of Korea; 3 Center for Research and Innovations, Adichunchanagiri University, Mandya, Karnataka India; 4 Department of Cellular and Genetic Medicine, School of Basic Medical Sciences, Fudan University, Shanghai, China

**Keywords:** Cell biology, Tumour angiogenesis

## Abstract

Vascular endothelial growth factor (VEGF) signaling is a critical regulator of vasculogenesis, angiogenesis, and lymphangiogenesis, processes that are vital for the development of vascular and lymphatic systems, tissue repair, and the maintenance of homeostasis. VEGF ligands and their receptors orchestrate endothelial cell proliferation, migration, and survival, playing a pivotal role in dynamic vascular remodeling. Dysregulated VEGF signaling drives diverse pathological conditions, including tumor angiogenesis, cardiovascular diseases, and ocular disorders. Excessive VEGF activity promotes tumor growth, invasion, and metastasis, while insufficient signaling contributes to impaired wound healing and ischemic diseases. VEGF-targeted therapies, such as monoclonal antibodies and tyrosine kinase inhibitors, have revolutionized the treatment of diseases involving pathological angiogenesis, offering significant clinical benefits in oncology and ophthalmology. These therapies inhibit angiogenesis and slow disease progression, but they often face challenges such as therapeutic resistance, suboptimal efficacy, and adverse effects. To further explore these issues, this review provides a comprehensive overview of VEGF ligands and receptors, elucidating their molecular mechanisms and regulatory networks. It evaluates the latest progress in VEGF-targeted therapies and examines strategies to address current challenges, such as resistance mechanisms. Moreover, the discussion includes emerging therapeutic strategies such as innovative drug delivery systems and combination therapies, highlighting the continuous efforts to improve the effectiveness and safety of VEGF-targeted treatments. This review highlights the translational potential of recent discoveries in VEGF biology for improving patient outcomes.

## Introduction

Vascular endothelial growth factors (VEGF) and their receptors (VEGFR) are critical factors of angiogenesis, a process critical for physiological and pathological conditions.^1,2^ Specifically, the signaling axis of VEGF-VEGFR controls endothelial cell (EC) proliferation, migration, and survival, thereby facilitating the organized development of new blood vessels. Vascular development is indispensable for embryonic growth, wound healing, and the female reproductive cycle.^1^ However, when VEGF signaling is dysregulated, various disorders such as cancer, diabetic retinopathy (DR, diabetes-induced retinal vascular disorder), and age-related macular degeneration (AMD, retinal disease characterized by macular degeneration) develop.^3^ Extensive research on VEGF-VEGFR signaling has advanced the development of therapeutic inhibitors that disrupt pathological angiogenesis. As a result, this therapeutic intervention has achieved significant milestones, especially in cancer therapy and ocular disease treatment. Despite this progress with anti-angiogenic drugs, therapeutic resistance and the complex regulation of angiogenesis remain major challenges, highlighting the critical need to target the VEGF-VEGFR pathway across various indications effectively.

**Fig. 1:**
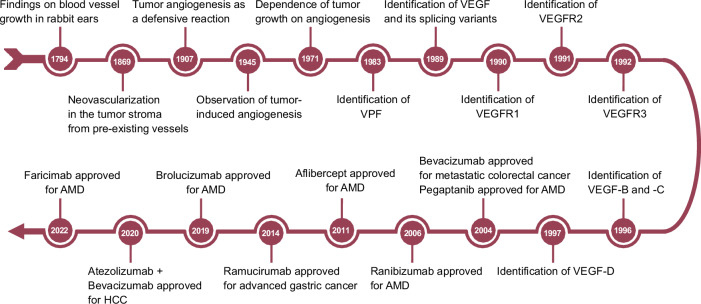
Timeline of key discoveries in angiogenesis research. The timeline begins with John Hunter’s observation of blood vessel growth and follows major advancements, including Folkman’s hypothesis, which links tumor growth to angiogenesis (1971). The significant milestones include the discovering the vascular permeability factor in 1983 and identifying vascular endothelial growth factor (VEGF), its receptors, and other VEGF family members between 1989 and 1997. It also highlights the approval of several anti-angiogenic drugs: pegaptanib for age-related macular degeneration (AMD) in 2004, bevacizumab for metastatic colorectal cancer in 2004, ranibizumab for AMD in 2006, aflibercept for AMD in 2011, ramucirumab for advanced gastric cancer in 2014, brolucizumab for AMD in 2019, the combination of atezolizumab and bevacizumab for hepatocellular carcinoma (HCC) in 2020, and faricimab for AMD in 2022. Created in BioRendender.com

The concept of angiogenesis has been acknowledged as a fundamental biological process since the 18th century (Fig. 1). British surgeon John Hunter first observed vascular growth in rabbit ears exposed to cold, with his 1794 publication laying the foundation for vascular research.^4,5^ Carl Thiersch later linked angiogenesis to tumor biology by demonstrating that new vessels in carcinoma originate from preexisting capillaries.^6^ Further expanding this understanding, the role of angiogenesis in cancer was emphasized by Rudolf Virchow and Ernst Goldmann, who proposed its dual function in tumor progression.^4,6^ This connection was further strengthened by Algire and Chalkley’s studies, which culminated in Judah Folkman’s 1971 hypothesis that tumor growth is angiogenesis-dependent, thereby establishing angiogenesis as a therapeutic target.^7^

A major breakthrough occurred in 1983 when Harold Dvorak and colleagues identified vascular permeability factor (VPF), a protein that significantly increased vascular permeability.^8^ Subsequent purification and sequencing confirmed that VPF was identical to VEGF,^8,9^ with Napoleone Ferrara successfully isolating VEGF from bovine pituitary follicular cells in 1989.^10^ This identification revealed VEGF as a potent endothelial mitogen with high specificity, further confirmed by studies on its role in vascular permeability and EC proliferation.^11,12^ The discovery of VEGF catalyzed the identification of additional family members, including VEGF-B, VEGF-C, and VEGF-D, and their receptors VEGFR1, VEGFR2, and VEGFR3.^13–18^ Moreover, alternative splicing of VEGF-A generated isoforms such as VEGF-A121, VEGF-A165, and VEGF-A189, which differ in receptor binding and bioavailability.^11,19^

The identification of VEGF as a central regulator of angiogenesis established its importance in both physiological and pathological contexts. Targeting VEGF has since become a cornerstone of anti-angiogenic therapies, profoundly impacting oncology and ophthalmology. This review examines VEGF family members and their receptors in angiogenesis and lymphangiogenesis, therapeutic interventions, and emerging research directions to advance precision medicine.

## Structural diversity of VEGF family members with their receptors

The VEGF family, comprising VEGF-A, VEGF-B, VEGF-C, and VEGF-D, regulates vascular and lymphatic development through isoforms generated by alternative splicing^20^ or proteolytic cleavage.^21^ These proteins share a cystine-knot motif critical for receptor binding and dimerization,^22,23^ interacting with VEGFR1, VEGFR2, VEGFR3, and neuropilin (NRP) as a co-receptor to modulate diverse functions.^24^

### VEGF-A

**Fig. 2:**
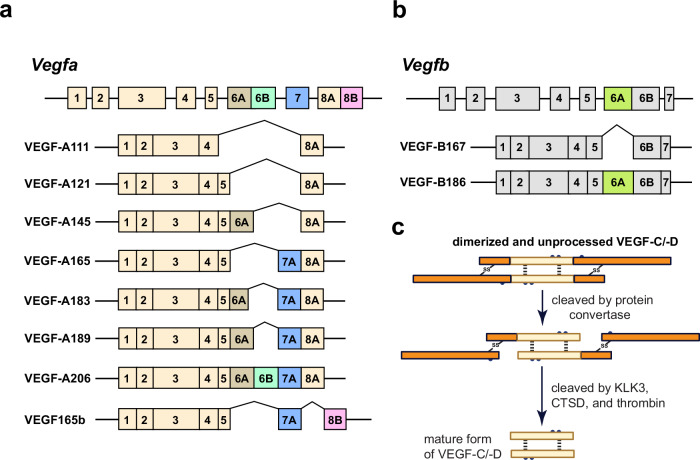
Schematic representation of alternative splicing variants and proteolytic processing of vascular endothelial growth factors (VEGFs). **a** VEGF-A isoforms generated by alternative splicing including VEGF-A111, VEGF-A121, VEGF-A145, VEGF-A165, VEGF-A183, VEGF-A189, VEGF-A206, and VEGF165b. Each isoform is represented by its exon composition, highlighting the variations in exons 6A, 6B, 7, and 8 across the isoforms contributing to differences in receptor-binding affinities and functional properties. **b** Alternative splicing generates two VEGF-B isoforms, VEGF-B167 and VEGF-B186. These isoforms differ in their use of either exon 6A or 6B, which determines their specific molecular characteristics. **c** VEGF-C and VEGF-D exist in unprocessed dimerized forms, and their proteolytic processing occurs through cleavage by the protein convertases, kallikrein 3 (KLK3), cathepsin D (CTSD), or thrombin, resulting in mature, active forms with modified functional properties. Created in BioRendender.com

VEGF-A exists in multiple isoforms (Fig. 2a), including VEGF-A111, 121, 145, 165, 183, 189, and 206, generated through alternative splicing of exons such as 6A, 6B, 7A, and 7B, which tailor their lengths and interactions with extracellular matrix (ECM) components to specific tissues and contexts.^24–26^ All isoforms share a conserved N-terminal region responsible for binding to VEGFRs and initiating signaling.^24^

VEGF-A111, excluding exons 5–7, diffuses easily, resists proteolysis, and is induced by DNA damage,^27^ while VEGF-A121, lacking exons 6A and 7, is soluble, forming stable homodimers resilient to oxidative stress.^19,25,28^ In contrast, VEGF-A145, missing exon 7, strongly binds ECM but exhibits reduced binding with its receptor and NRP1 due to missing motifs.^29,30^ VEGF-A165, the predominant isoform, features an N-terminal receptor-binding domain (RBD) and a C-terminal heparin-binding domain (HBD), enabling ECM retention and systemic distribution.^31–35^ This isoform binds VEGFR2 with a dissociation constant (Kd) of 1–10 nM, ensuring rapid receptor engagement at physiological levels,^24,36^ and forms a homodimer (Fig. 4a), with each monomer stabilized by hydrogen bonds and hydrophobic contacts, notably Glu64 and Asp63, interacting with the VEGFR2 D2 domain.^37–39^ The HBD of VEGF-A165 anchors it to the ECM, prolonging receptor activation and enhancing vascular stability.^40^

However, VEGF-A121, lacking this HBD (Fig. 2a), disperses widely but is unable to bind co-receptor, NRP1, resulting in lower VEGFR2 activation and approximately 10-100 times reduced mitogenic activity, often leading to leakier vessels due to limited tissue retention.^30,41,42^ Similarly, VEGF-A145 retains partial ECM-binding capacity but has reduced heparin affinity compared with VEGF-A189, forming intermediate gradients that support vascularization when VEGF-A165 is insufficient.^29^ VEGF-A183, with a truncated exon 6A, exhibits intermediate heparin-binding affinity, remaining ECM-associated,^43,44^ whereas VEGF-A189 and VEGF-A206, both retaining exons 6 and 7 (Fig. 2a), tightly bind heparan sulfate proteoglycans (HSPGs), restricting diffusion and forming steep VEGF gradients that promote dense, structured vascular networks.^25,45^ Furthermore, VEGF-A189’s interaction with NRP1 enhances VEGFR2 signaling, although proteolytic cleavage modulates its bioavailability.^46^ Lastly, VEGF-A206, the longest isoform, contains an extended HBD, strongly binding the ECM to localize angiogenic activity, particularly in fetal and pathological tissues.^20,25,47^ Thus, the diverse isoforms of VEGF-A, through their unique structural properties and receptor interactions, finely tune angiogenesis across physiological and pathological contexts.

### VEGF-B

VEGF-B exists in two isoforms, VEGF-B167 and VEGF-B186 (Fig. 2b), arising from alternative splicing and distinguished by their C-terminal domains.^14^ Both share an identical N-terminal domain containing a VEGF homology domain (VHD) and eight cysteine residues crucial for forming intra- and intermolecular disulfide bonds, predominantly resulting in homodimers with a molecular weight of 44–54 kDa, widely expressed in normal and tumor tissues.^14,48^ Specifically, VEGF-B167, featuring a heparin-binding C-terminal domain (CTD), attaches to cell surface heparan sulfate proteoglycans (HSPGs) and constitutes >80% of VEGF-B levels in most tissues.^49^ In contrast, VEGF-B186, with a hydrophobic O-glycosylated CTD, is freely soluble, upregulated in several primary tumors and tumor cell lines, and prone to proteolytic cleavage that generates biologically active fragments.^50^ Additionally, both isoforms can form disulfide-linked heterodimers with VEGF-A, which remain cell-associated, unlike the secreted VEGF-A homodimers, suggesting diverse functions across physiological contexts.^14,51^ Functionally, VEGF-B binds exclusively to VEGFR1 (Fig. 4a), engaging the same region as VEGF-A but with slightly lower affinity (Kd 1–3 nM for VEGF-B167), and plays a specialized role in tissue protection and metabolic regulation rather than promoting angiogenesis.^52–57^ However, despite this high-affinity binding, VEGF-B167 induces minimal signaling due to low kinase activity of VEGFR1, constrained by an inhibitory sequence in the juxtamembrane region, thus limiting its ability to transduce angiogenic signals.^52,58,59^ Consequently, unique structural and binding properties of VEGF-B underscore its distinct contributions to tissue homeostasis and disease.

### VEGF-C

VEGF-C stands out in the VEGF family for its structural complexity and intricate post-translational modifications.^15^ It is a 419-amino acid protein with 30% sequence identity to VEGF-A and 27% to VEGF-B.^15,60^ VEGF-C is initially synthesized as an inactive precursor sequestered in the ECM (Fig. 2c) and undergoes sequential proteolytic cleavages to achieve full activation.^61^

Proprotein convertases (PC) including furin generate a partially active intermediate, while the final cleavage by ADAMTS3 produces the mature form (~21 kDa) with high affinity for VEGFR2 and VEGFR3, driving lymphangiogenesis and vascular remodeling.^62,63^ Additionally, proteases, such as plasmin, thrombin, KLK3, and cathepsin D, modify VEGF-C, altering its receptor-binding affinities and activity profiles.^61,64^ These modifications, particularly in the tumor microenvironment (TME), fine-tune the role of VEGF-C in cancer progression, metastasis, and tumor-associated lymphangiogenesis.

### VEGF-D

VEGF-D was identified from a human EST sequence, resulting in a full-length cDNA encoding a 354-amino acid protein with 23% identity to VEGF-C.^16^ It is structurally similar to VEGF-C but features unique N- and C-terminal extensions. VEGF-D undergoes proteolytic processing (Fig. 2c) to generate isoforms with distinct receptor affinities.^65,66^ The unprocessed precursor (~50 kDa) is inactive until N- and C-terminal propeptides are cleaved. Intermediate forms (31–35 kDa) bind VEGFR3 with high affinity but exhibit lower activity than the fully processed form (~21 kDa), which binds VEGFR2 and VEGFR3, promoting angiogenesis and lymphangiogenesis. This maturation, regulated by extracellular proteases such as PC and plasmin, modulates receptor specificity and bioactivity.^65,67^ VEGF-D differs from VEGF-C in expression patterns, predominantly found in the lungs, heart, skeletal muscle, and intestines.^67,68^ VEGF-C and VEGF-D regulate lymphangiogenesis via VEGFR3 and contribute to angiogenesis through VEGFR2 under specific conditions.^69,70^ This dual functionality highlights its role in coordinating vascular and lymphatic growth, particularly in cancer metastasis, underscoring its significance in tissue homeostasis and disease progression.

VEGF-C and VEGF-D primarily drive lymphangiogenesis by binding to VEGFR3 (Fig. 4a), a receptor predominantly expressed in lymphatic endothelial cells (LECs).^71^ Both ligands require proteolytic processing for full activity. Initially, full-length VEGF-C exhibits low affinity for VEGFR3, but cleavage by enzymes such as ADAMTS3 (Fig. 2c) enhances its binding strength, potently activating VEGFR3 to promote LEC proliferation and migration.^15,72^ Similarly, VEGF-D binds both VEGFR2 and VEGFR3 (Fig. 4a), with its affinity increasing post-processing, enabling the mature form to strongly support angiogenesis and lymphangiogenesis, while the unprocessed form binds VEGFR3 less effectively.^66,67,73^ Additionally, VEGF-D can activate VEGFR2/VEGFR3 heterodimers, contributing to lymphatic dilation and vascular remodeling, particularly in cancer.^74^

### VEGFxxxb

VEGF-A isoforms are primarily pro-angiogenic; however, specific inhibitory variants, known as VEGFxxxb isoforms,^75^ such as VEGF165b, arise through alternative splicing at the distal splice site of exon 8 (Fig. 2a). These isoforms feature a C-terminal sequence (SLTRKD) that replace the pro-angiogenic CDKPRR sequence in VEGF-Axxx isoforms,^76^ allowing them to bind VEGFR2 competitively without initiating complete receptor phosphorylation and signaling, which reduce angiogenic responses.^77^

The existence and role of VEGFxxxb isoforms in angiogenic balance are debated. Some studies suggest they may be methodological artifacts, as large-scale RNA-seq often fails to detect them in human tissues, and antibody specificity is questioned.^78–81^ This inconsistency fuels skepticism, implying VEGFxxxb isoforms might only appear under specific experimental conditions. However, VEGF-A165b, known for its anti-angiogenic properties, is elevated in peripheral artery disease (PAD) and linked to reduced vascularization despite higher total VEGF-A levels.^82^ In a PAD mouse model, metabolic dysfunction and Wnt5a signaling increased VEGF-A165b, impairing angiogenesis, while its neutralization restored revascularization. These results indicate VEGF-A165b may suppress pathological angiogenesis in PAD, suggesting potential as a therapeutic target. While the physiological relevance of VEGFxxxb remains controversial, the evidence of VEGF-A165b’s inhibitory effect in PAD highlights their potential clinical significance.

### Placenta growth factor (PlGF)

PlGF is a VEGF family glycoprotein sharing ~42% sequence homology with VEGF-A.^83,84^ It features a conserved VHD essential for dimerization and receptor binding. Alternative splicing of PlGF generates four isoforms: PlGF-1, PlGF-2, PlGF-3, and PlGF-4, distinguished by C-terminal variations that affect their receptor affinities, spatial distribution, and biological properties.^85–87^

PlGF-1 (131 amino acids) is the most studied isoform, lacking HBD and circulating as a soluble protein. It forms homo- and heterodimers with VEGF family members to modulate VEGF-A activity.^83,88^ PlGF-2 (152 amino acids) contains an additional 21-amino acid HBD, enabling interactions with the ECM and cell surface, confining its distribution and enhancing localized paracrine signaling.^85,89^ PlGF-3 (203 amino acids) includes a unique 72-amino acid C-terminal region, with tissue-specific roles yet to be fully defined, suggesting potential regulatory functions in specific pathological contexts.^86^ In addition, PlGF-4 (224 amino acids) is the longest isoform, similar to PlGF-2 in HBD-mediated ECM and cell interactions, although expressed at lower levels.^90^ Notably, the VEGF-A/PlGF heterodimer primarily binds to VEGFR1, which leads to enhanced receptor activation compared with the individual factor alone,^88^ thereby promoting EC migration, permeability, particularly in pathological conditions, such as cancer and ischemia, where it facilitates neovascularization and vascular inflammation.^53^

PlGF binds exclusively to VEGFR1 (Fig. 4a), playing a significant role in pathological angiogenesis.^91^ Through high-affinity binding, it promotes VEGFR1 dimerization and activation,^88^ triggering unique angiogenic and inflammatory signaling pathways despite the lower kinase activity of VEGFR1.^53^ PlGF modulates VEGF-A signaling by competing for VEGFR1 binding, reducing VEGF-A sequestration.^92^ This competition shifts receptor dynamics, freeing more VEGF-A to engage VEGFR2, thereby amplifying VEGF-A-driven angiogenesis. Furthermore, PlGF enhances vascular remodeling and inflammation through VEGFR1 signaling, extending its effect beyond direct receptor activation.

### VEGF receptors

#### VEGFR1

**Fig. 3:**
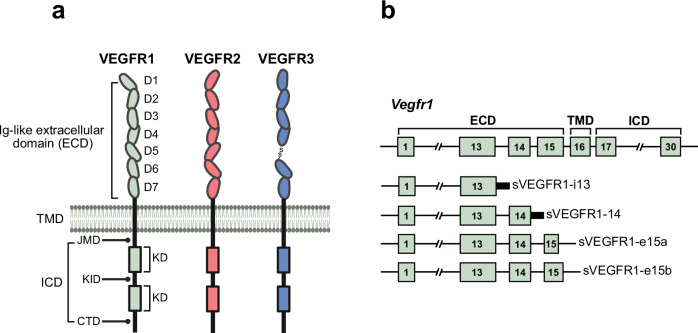
Structural domains of vascular endothelial growth factor receptors (VEGFRs) and isoforms of soluble VEGFR1. **a** Domain organization of VEGFR1, VEGFR2, and VEGFR3. Each receptor has key structural features, including Ig-like extracellular domains (D1-D7), transmembrane domain (TMD), juxtamembrane domain (JMD), kinase domain (KD), and C-terminal domain (CTD). **b** Alternative splicing generates five distinct splice variants of VEGFR1: the full-length VEGFR1, VEGFR1 with intron 13 retention (sVEGFR1-i13), VEGFR1 with intron 14 retention (sVEGFR1-i14), and the soluble isoforms sVEGFR1-e15a and sVEGFR1-e15b with alternative terminal exons. The schematic highlights the differences in domain configurations between full-length receptors and their soluble forms, illustrating the structural diversity resulting from alternative splicing. Created in BioRendender.com

VEGFR1, also known as Fms-like tyrosine kinase-1 (Flt1),^93^ regulates various aspects of angiogenesis through its complex multi-domain structure, serving as a decoy receptor.^94^ Its extracellular domain (ECD) comprises seven immunoglobulin-like (Ig) domains, with D2 and D3 forming the primary ligand-binding interface that confers high affinity for VEGF-A (Fig. 3a). This interaction sequesters VEGF-A, reducing its availability for VEGFR2, and thus modulates angiogenic signaling.^54,95^ The remaining Ig-like domains (D4-D7) contribute to structural integrity and facilitate receptor dimerization, supporting interactions with other receptors and stabilizing the receptor complex.^96–99^

The transmembrane domain (TMD) is crucial for anchoring the receptor to the cell membrane and facilitating its function in VEGF signaling.^100^ Notably, VEGFR1 is expressed in two isoforms: a membrane-bound full-length form and a soluble variant lacking the TMD.^94^ The TMD anchors VEGFR1 to the cell surface, enabling it to modulate the spatial distribution of VEGF and regulate downstream signaling.^101^ The intracellular domain (ICD) of VEGFR1 (Fig. 3a) comprises the juxtamembrane domain (JMD), split tyrosine kinase domain (KD), and C-terminal tail. VEGFR1 exhibits low kinase activity compared with VEGFR2, which is linked to unique structural features. The JMD includes a repressor sequence with three serine residues that inhibit downstream signaling, such as PI3K activation, even in the presence of VEGF.^59^ Additionally, an “electrostatic latch” in the JMD maintains VEGFR1 in an auto-inhibitory conformation, preventing ligand-independent activation.^102^ Moreover, VEGFR1 catalytic function is impaired by a critical asparagine in its activation loop, replacing a conserved aspartic acid, which disrupts transphosphorylation of essential tyrosine residues, further limiting its activity. These structural elements collectively define its unique role in signaling.^103,104^ Collectively, the complex architecture and regulatory features of VEGFR1 enable it to fine-tune angiogenesis by modulating VEGF availability and selectively suppressing signaling.

#### Soluble VEGFR1 (sVEGFR1)

The sVEGFR1 isoforms, sVEGFR1-i13, sVEGFR1-i14, sVEGFR1-e15a, and sVEGFR1-e15b (Fig. 3b), arise from alternative splicing and retain VEGF-binding capacity without downstream signaling.^105^ Produced by various cells, including endothelial and melanoma cells, sVEGFR1 often circulates bound to VEGF, playing a key role in regulating angiogenesis, particularly in pathological states such as pre-eclampsia.^106^

sVEGFR1-i13, the most common isoform, is generated by intron 13 retention^107^ and acts as a VEGF scavenger, inhibiting VEGF-A-induced angiogenesis by forming non-signaling complexes with VEGFR2.^108^ sVEGFR1-i14, generated via intron 14 retention (Fig. 3b), is primarily expressed in the brain and testes,^109^ where it regulates local VEGF activity and vascular stability. sVEGFR1-e15a, formed through exon 15a inclusion, is abundant in the placenta, playing a crucial role in vascular development.^105,109^ Elevated levels are linked to pre-eclampsia, disrupting angiogenesis. Moreover, sVEGFR1-e15b, produced by exon 15b inclusion (Fig. 3b), features matrix-binding properties and is expressed in the placenta and vascular tissues, contributing to abnormal angiogenesis in cancers and ocular diseases.^105^ Functioning as decoy receptors, these isoforms regulate VEGF ligand distribution, limiting VEGFR2 activation and attenuating pro-angiogenic signaling. Collectively, these variants provide a dynamic regulatory mechanism that ensures tissue-specific vascular stability and growth control.

#### VEGFR2

VEGFR2, also known as kinase insert domain receptor (KDR) in humans and fetal liver kinase-1 (Flk1) in mice, is a receptor tyrosine kinase (RTK).^110–112^ The ECD comprises seven Ig-like domains (D1-D7), each with D2 and D3 primarily responsible for high-affinity VEGF binding (Fig. 3a). D2 forms hydrophobic interactions as the main binding site, while D3 stabilizes the receptor-ligand complex through hydrophilic interactions.^113,114^ Ligand binding induces conformational changes in D2 and D3, essential for receptor dimerization and activation.^113,115^ D4 and D5 support receptor dimerization and allosteric regulation, ensuring proper alignment of the intracellular kinase domains,^116^ while D7 facilitates receptor dimerization via charged interactions in its E-F loop.^116,117^ Ligand binding also reorients TMD helices, a prerequisite for the kinase domain activation.^118^ Although VEGFR2 can dimerize, ligand binding ensures correct helix alignment downstream signaling.^119^

The JMD functions as a regulatory gate, keeping the receptor in an inactive state until ligand-induced conformational changes activate the KD.^100^ The KD phosphorylates specific tyrosine residues, such as Tyr1059 (Y1059) in the activation loop, transitioning the kinase to an active state.^120^ The kinase insert domain (KID) serves as a docking platform for downstream signaling molecules, with phosphorylation at sites such as Y951 enabling interactions with effectors such as TSAd, which mediate vascular permeability and remodeling.^121^ The CTD stabilizes the receptor in an inactive state but undergoes structural rearrangements upon ligand binding to facilitate full activation. Phosphorylation of key residues within the CTD, such as Y1175, recruits signaling proteins including PLCγ1, driving EC proliferation and migration.^122^ Moreover, the KID and CTD are integral to intrinsic receptor dimerization, which is essential for full kinase activation.^123^ Deletion of these domains diminishes dimerization efficiency and compromises signaling. Together, these structural features ensure precise regulation of VEGFR2 activity, supporting its pivotal role in vascular development and pathological angiogenesis.

#### VEGFR3

VEGFR3, also known as Fms-related tyrosine kinase-4, is an RTK essential for vascular development but is primarily involved in lymphangiogenesis. A unique feature of VEGFR3 is the proteolytic cleavage of its ECD (Fig. 3a), resulting in a mature form linked by disulfide bonds.^124^ D1-D3 in the ECD are critical for VEGF-C binding, particularly through interactions with D2, while D1 stabilizes the ligand-receptor complex. D4-D7 mediate receptor dimerization and activation.^67,124^ Proteolytic cleavage at D5 divides the ECD into two subunits, which remain connected by disulfide bonds. This cleavage is essential for effective ligand binding, signaling, and receptor activation, facilitated by homotypic interactions between D5 and D7.^124^ The TMD and ICD are structurally similar to those of VEGFR1 and VEGFR2, featuring a single-pass transmembrane region and split tyrosine KD, enabling ligand-induced dimerization and autophosphorylation.^125^ VEGFR3 primarily mediates lymphangiogenesis through VEGF-C and VEGF-D signaling in lymphatic endothelial cells (LECs). It also modulates VEGFR2 activity via heterodimerization, indirectly supporting blood vessel development while maintaining its distinct role in lymphatic growth.

#### VEGFR dimerization

**Fig. 4:**
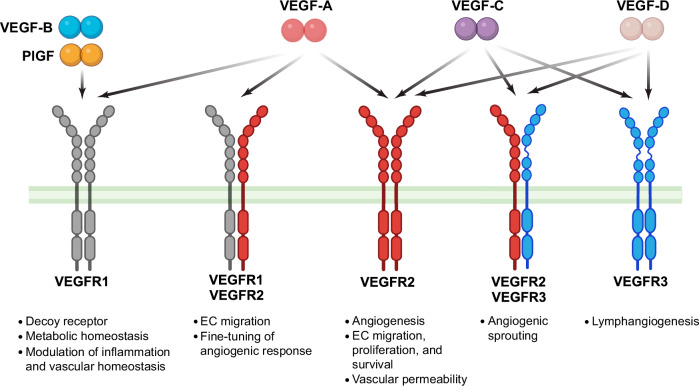
Vascular endothelial growth factor (VEGF) family members and their receptors. Binding of VEGF-A, -B, -C, -D, and PlGF with their respective receptors, VEGFR1, VEGFR2, and VEGFR3, highlighting the primary receptor specificities. VEGF-A binds VEGFR1 and VEGFR2, VEGF-B and PlGF selectively interact with VEGFR1. VEGFR2 is the primary mediator of VEGF-A-driven angiogenesis, also binding VEGF-C. VEGFR3 predominantly binds VEGF-C and VEGF-D, regulating lymphangiogenesis. This binding schematic illustrates the selective affinities of VEGF family members for their receptors and their distinct roles in vascular and lymphatic regulation. Additionally, VEGF-B is depicted as a metabolic regulator that acts through VEGFR1, while PlGF is shown to modulate inflammation and vascular homeostasis. Created in BioRendender.com

VEGFR homodimers are formed when two identical receptors pair in response to ligand binding. VEGFR2 homodimers (Fig. 4) are mainly activated by VEGF-A, which triggers a series of key processes in ECs.^126,127^ Upon activation, these receptors undergo autophosphorylation, initiating intracellular signaling cascades, which drive angiogenesis.^126^ In contrast, VEGFR1 homodimers, which are capable of binding to VEGF-A and other ligands, play a regulatory role. They regulate VEGFR2 signaling and inhibit excessive angiogenic responses.^127^ VEGFR2 can exist in a monomeric or dimeric state without ligand binding.^128,129^ VEGFR2 forms dimers without ligands at physiological levels, showing low levels of basal phosphorylation.^129^ This pre-formed, partially active state enables rapid angiogenic signaling, offering a refined model of RTK activation beyond traditional ligand-induced dimerization.

Heterodimerization fine-tunes VEGF signaling by modulating receptor activity in response to ligand concentrations and receptor abundances. VEGFR1-VEGFR2 heterodimers (Fig. 4) regulate VEGF-A activity by inhibiting VEGFR2, primarily through reduced PI3K-mediated phosphorylation, balancing EC migration and angiogenic processes.^130^ VEGFR2-VEGFR3 heterodimers, induced by VEGF-C and VEGF-D (Fig. 4), are critical for blood and lymphatic vessel formation, particularly in angiogenic tip cells where they control sprouting and branching.^131,132^ Modeling studies predict that VEGFR1-VEGFR2 heterodimers constitute 10–50% of active signaling complexes, depending on receptor expression levels. High VEGFR2 abundance suppresses VEGFR1 homodimer formation, favoring VEGFR2 signaling.^128^ Notably, VEGFR1-VEGFR2 heterodimers are more prevalent in neuronal cells, whereas VEGFR2 homodimers dominate in ECs.^133^ This differential dimerization may explain VEGF’s distinct roles in nerve regeneration versus blood vessel growth, underscoring the various regulatory functions of receptor interactions across physiological contexts.

**Fig. 5:**
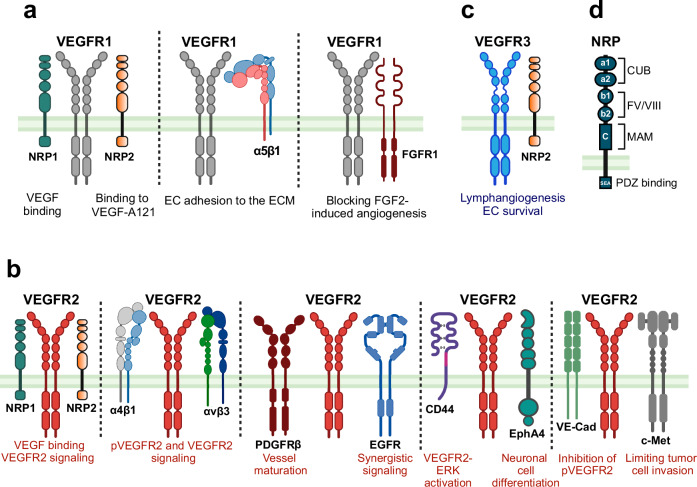
Interactions of vascular endothelial growth factor receptors (VEGFR) with co-receptors and structural features of Neuropilin. **a** Interaction of VEGFR1 with co-receptors NRP1 and NRP2, α5β1 integrin, and FGFR1. The VEGFR1-NRP2 interaction facilitates VEGF-A121 binding, whereas VEGFR1 engagement with α5β1 promotes EC adhesion to the extracellular matrix. Additionally, the interaction of VEGFR1 with FGFR1 blocks FGF2-induced angiogenesis, highlighting the role of the VEGFR1 complex in modulating angiogenic signaling pathways and EC behavior. **b** Association of VEGFR2 with various co-receptors and adhesion molecules, including NRPs (NRP1 and NRP2), PDGFRβ, EGFR, CD44, EphA4, VE-Cadherin, and c-Met. These interactions contribute to the specificity and complexity of VEGF signaling, enabling crosstalk between VEGF receptors and other signaling pathways. Such an association with co-receptors and adhesion molecules enhances cellular responses, including EC migration, adhesion, and survival, supporting key physiological processes such as angiogenesis, vascular maturation, and cellular adhesion dynamics within the VEGF pathway. **c** Interaction between VEGFR3 and the co-receptor NRP2, which plays a critical role in promoting lymphangiogenesis and supporting EC survival. **d** NRP is shown with additional structural motifs, including the CUB, FV/VIII, MAM, and SEA domains. These domains contribute to receptor interactions and enhance signaling specificity within the VEGF pathway, potentially influencing binding affinities and receptor-ligand selectivity. NRP neuropilin, FGFR fibroblast growth factor receptor, PDGFR platelet-derived growth factor receptor, EGFR epidermal growth factor receptor. Created in BioRendender.com

Heteromeric complexes between VEGFRs and non-VEGFRs generate diverse signaling outcomes, enhancing functional flexibility. VEGFR1 forms heterodimers with NRP1 (Fig. 5a), increasing VEGF binding and modulating signaling.^134^ Additionally, VEGFR1 also forms a complex with NRP2 (Fig. 5a), enabling NRP2 to bind VEGF-A121.^135^ Moreover, VEGFR1 interacts with integrin α5β1 (Fig. 5a) to mediate endothelial adhesion to the ECM^136^ and binds FGFR1, suppressing FGF2-induced angiogenesis.^137^

VEGFR2 similarly forms complexes with non-VEGFRs, notably NRP1 and NRP2,^138^ which act as co-receptors (Fig. 5b). VEGFR2-NRP1 heterodimers enhance VEGF binding and signaling potential. VEGF facilitates these interactions by bridging VEGFR2 and NRP1, forming complexes in *cis* (within the same cell) or *trans* (between cells), with the *cis* configuration being kinetically favored.^139,140^ The VEGF-NRP1 interaction relies on the C-terminal tail of VEGF binding to the b1 domain of NRP1, offering structural flexibility that allows these complexes to adapt to diverse cellular contexts.

αvβ3 integrin binds vitronectin to amplify VEGFR2 phosphorylation and angiogenic signaling. Similarly, α4β1 integrin forms a complex with VEGFR2 in chronic lymphocytic leukemia cells (Fig. 5b), enhancing VEGF signaling and inducing apoptosis when disrupted,^141^ underscoring a therapeutic target. VEGFR2-platelet-derived growth factor receptor β (PDGFRβ) heterodimers (Fig. 5b) link angiogenesis to vessel maturation via pericyte recruitment, stabilizing tumor vasculature.^142^ VEGFR2-epidermal growth factor receptor (EGFR) heterodimers, formed in the presence of both EGF and VEGF, offer a ligand-dependent mechanism for diversifying cancer signaling.^143^ Moreover, the CD44v6 isoform acts as a co-receptor for VEGFR2 in ECs,^144^ enhancing ERK signaling (Fig. 5b). EphA4, a receptor tyrosine kinase involved in neuronal development, forms a kinase-dependent complex with VEGFR2 (Fig. 5b), promoting neuronal differentiation in neural stem and progenitor cells when activated by ephrin A1 and VEGF-A165.^145^

VEGFR2 forms a complex with VE-cadherin (VE-cad) at cell-cell junctions (Fig. 5b), limiting VEGFR2 phosphorylation by recruiting phosphatases.^146^ This interaction reduces VEGF-induced proliferation while promoting EC survival. Additionally, VEGFR2 interacts with c-Met (hepatocyte growth factor receptor) in a heterocomplex (Fig. 5b), where VEGF-A recruits protein tyrosine phosphatase 1B (PTP1B) to dephosphorylate c-Met, inhibiting its signaling and reducing tumor cell migration and invasiveness.^147^ VEGFR3 also forms a complex with NRP2 (Fig. 5c), enhancing lymphangiogenesis and supporting EC survival.^148^ VEGF-C stimulation strengthens this interaction, leading to increased VEGFR3 phosphorylation and activation of downstream signaling. This association with non-VEGFRs highlights the complex regulation of angiogenesis and lymphangiogenesis, offering new avenues for therapeutic intervention for abnormal blood and lymphatic vessel formation.

### Neuropilin

NRP1 and NRP2 are non-tyrosine kinase co-receptors that enhance VEGF signaling by binding to the VEGF family in conjunction with VEGFRs.^149^ Although they lack catalytic activity, their ECD enhance VEGF-VEGFR signaling, playing critical roles in angiogenesis and lymphangiogenesis.

Both NRP1 and NRP2 feature a structured ECD comprising two complement-binding (CUB) domains (a1/a2) and two coagulation factor homology domains (b1/b2), which enable their binding ligands such as VEGFs and semaphorins (Fig. 5d). The a1/a2 domains are essential for binding semaphorins, such as sema3A, involved in axonal guidance, while the b1 domain allows high-affinity binding to VEGF-A165 in NRP1 and to VEGF-C in NRP2, with the b2 domain enhancing these interactions.^140^ Additionally, both NRPs contain a MAM domain (c) that supports homodimerization and receptor interactions, strengthening their signaling roles.^150^ Although both have short ICDs without catalytic activity, they feature PDZ-binding motifs (SEA) that allow interactions with intracellular proteins, such as synectin, to facilitate downstream signaling.^151^ NRP2 uniquely includes soluble splice variants (sNRP2) that act as decoy receptors by sequestering VEGF-C and VEGF-D, thus modulating their bioavailability and lymphangiogenic functions.^152^ Together, the structural motifs of NRP1 and NRP2 enable their versatile roles across physiological and pathological processes.

## VEGFR and NRP signaling pathways

### VEGFR1 signaling

**Fig. 6:**
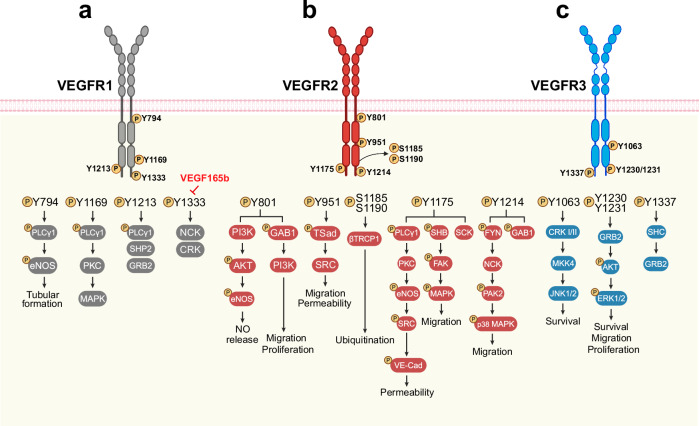
Signal transduction of vascular endothelial growth factor receptors. **a** VEGFR1: Schematic representation of critical phosphorylation sites and their roles in signaling. Y794 in the juxtamembrane domain facilitates PLCγ1 binding and eNOS activation, promoting nitric oxide (NO) release and tubular structure formation. Y1169 recruits PLCγ1, while Y1213 enables binding of SH2 domain proteins such as PLCγ1, SHP2, and GRB2, supporting PI3K-mediated cell survival and proliferation. Y1242 and Y1333 support PLCγ1 phosphorylation but are insufficient for full mitogenic signaling. VEGF165b inhibits Y1333 phosphorylation, suppressing VEGFR1-STAT3 signaling, which can be restored to enhance angiogenesis in peripheral artery disease. **b** VEGFR2: Key phosphorylation sites and their roles in vascular functions. Y801 and Y1214 activate GAB1, promoting PI3K/AKT signaling, endothelial survival, migration, and NO production. Y951 (Y949 in mice) binds TSAd, regulating vascular permeability via the VEGFR2-TSAd-SRC complex. Y1054 and Y1059 are essential for full kinase activity, while Y1175 recruits adaptor proteins such as SHB and PLCγ1. Serine phosphorylation at S1183 and S1188 in mice (S1185 and S1190 in humans) promotes VEGFR2 degradation via β-TRCP1-mediated ubiquitination, regulating receptor stability. **c** VEGFR3: Schematic representation of essential phosphorylation sites and their roles in cell signaling. Phosphorylation at Y1063 recruits CRK I/II, activating the JNK pathway to support cell survival. Y1230 and Y1231 facilitate GRB2 recruitment, initiating ERK and AKT signaling pathways that promote cell proliferation and migration. Y1337 acts as a docking site for the GRB2-SHC complex, driving RAS-mediated signaling. PLCγ1 Phospholipase C gamma 1, GAB1 GRB2-associated binding protein 1, SHP2 Src homology-2 domain-containing protein tyrosine phosphatase-2, GRB2 growth factor receptor-bound protein 2, PI3K Phosphoinositide 3-kinase, TSad T cell-specific adapter protein, SHC SH2 domain protein C1, SHB SH2 domain-containing adapter protein B, SCK SHC-like protein, CRK I/II CT10 regulator of kinase I and II. Created in BioRendender.com

Specific tyrosine phosphorylation sites on VEGFR1 are crucial for recruiting adaptor molecules and mediating key signaling pathways to regulate cell migration, proliferation and survival. In particular, Y794 is located in the JMD region (Fig. 6a) and is an important site for PLCγ1 binding.^153^ Additionally, activation of endothelial nitric oxide synthase (eNOS) depends on Y794, resulting in nitric oxide (NO) release, which is essential for forming capillary-like networks in vitro.^154^ Y1169 was identified as a major phosphorylation site,^153,155^ critical for recruiting PLCγ1. Y1213, Y1242, and Y1333 were identified as key phosphorylation sites for VEGFR1’s biological functions (Fig. 6a). Phosphorylation at Y1213 enables the binding of several SH2 domain-containing proteins, such as PLCγ1, SHP2, and GRB2. Specifically, PLCγ1 and SHP2 were found to be directly associated with Y1213 in a phosphotyrosine-dependent manner.^156^ They also facilitate the binding of PI3K, which play an important role in cell proliferation and survival in response to VEGF.^157^ Although Y1242 and Y1333 retain kinase activity and support PLCγ1 phosphorylation, they cannot mediate downstream mitogenic signals.^156,158^ Y1333 also binds important signaling proteins such as NCK and CRK, while it allows for PLCγ1 recruitment, it is insufficient for full signal transduction and biological responses such as cell proliferation.^156,158^ Interestingly, phosphorylation at Y1333 was inhibited by VEGF165b in ischemic muscle and blocking VEGF165b restored VEGFR1 phosphorylation and enhanced the VEGFR1-STAT3 pathway, promoting angiogenesis and perfusion recovery in PAD.^159^

Although VEGF-B binds to VEGFR1 with high affinity,^160^ it does not induce tyrosine phosphorylation.^55^ However, in human retinal ECs, it leads to only a modest increase in VEGFR1 phosphorylation.^161^ This contrasts with other VEGF family ligands such as VEGF-A, which robustly activate VEGFR1, triggering extensive phosphorylation at multiple tyrosine residues. Mass spectrometry analysis revealed that stimulation with human PlGF-2 resulted in Y1309 phosphorylation of VEGFR1 whereas Y1213 remained unphosphorylated,^162^ highlighting a selective phosphorylation pattern in response to different ligands that could lead to the differential signaling potential of VEGF-B and PlGF on VEGFR1.

### sVEGFR1 signaling

sVEGFR1 is highly expressed in trophoblasts, particularly under hypoxic conditions, it reduces VEGF-mediated EC migration and angiogenesis. This upregulation, linked to conditions such as pre-eclampsia, correlates with impaired placental angiogenesis.^163^ Additionally, VEGF-A stimulates its own negative regulator, sVEGFR1, through the VEGFR2-PKC-MEK pathway, establishing a feedback loop that limits VEGF-A activity and angiogenesis.^164^

However, in squamous cell lung carcinoma, sVEGFR1-i13 regulates a β1 integrin/VEGFR autocrine loop, promoting tumor proliferation and resistance to anti-angiogenic therapies.^165^ Elevated sVEGFR1-i13 levels are associated with advanced disease stages and poor outcomes. Moreover, sVEGFR1 interacts with α5β1 integrins, modulating cytoskeletal dynamics, and activating RAC1 signaling, thereby driving EC migration and motility through the phosphorylation of proteins such as MARCKS and RADIXIN. Notably, sVEGFR1 is essential for podocyte function and glomerular barrier integrity, interacting with lipid microdomains to regulate cytoskeletal organization and actin dynamics.^166^ Moreover, loss of sVEGFR1 disrupts the cytoskeleton, leading to proteinuria and glomerular dysfunction. These findings highlight the dual functions of sVEGFR1, acting as both an inhibitor and promoter of angiogenesis depending on the biological context and its molecular interactions.

### VEGFR2 signaling

VEGFR2 dimerizes and autophosphorylates upon VEGF binding, activating pathways that regulate EC proliferation, migration, and vascular permeability. Activation of VEGFR2 is characterized by significant heterogeneity at the single-molecule level, with receptor mobility and interactions varying across the EC surface.^167^ VEGF binding triggers diverse activation mechanisms, including ligand-induced dimerization and engagement with pre-formed dimers. Even without VEGF, VEGFR2 exists as dimers with low phosphorylation levels, with ligand binding enhancing kinase activity through conformational change.^129^ Structural studies emphasize inter-domain contacts in stabilizing dimer formation, but pathogenic mutations such as C482R and R1051Q disrupt regulation,^168^ causing ligand-independent activation and altered membrane dynamics, underscoring the complexity of VEGFR2 regulation and its implications for signaling and disease progression.

Kinase assays and phosphopeptide mapping analysis reveal VEGFR2 contains 19 tyrosine residues, with 11 potential phosphorylation sites in non-catalytic regions.^169,170^ Key phosphorylation sites include Y951 in the KID and Y1054, Y1059, Y1175, and Y1214 in the C-terminal tail (Fig. 6b), while other residues, such as Y801, Y822, Y938, and Y996 remain unphosphorylated. Low-level phosphorylation at Y1305, Y1309, and Y1319 suggest minor role or transient roles. Phosphorylation at Y801 and Y1214, activates GAB1,^170^ promoting PI3K/AKT signaling for EC survival, migration, and NO production. Y951 (Y949 in mice) phosphorylation recruits TSAd, essential for vascular permeability by destabilizing VE-cad via the VEGFR2-TSAd-SRC complex.^169^ Mice with the Y949 mutation (*Flk1*^*Y949F/Y949F*^) show reduced VEGFA-induced vascular leakage and metastasis, offering therapeutic potential for conditions such as oxygen-induced retinopathy.^171^

Y1054 and Y1059 in the kinase activation loop are critical for full VEGFR2 activity, with mutations at these sites impairing downstream signaling.^172^ Y1175 recruits adaptor proteins such as SHB (SRC homology 2 domain-containing adaptor protein B), SCK (Shc-like protein), PLCγ1, and Y1173F mutations (*Flk1*^*Y1173F/+*^; Y1175 in humans) in mice reduce vascular leakage and improve chemotherapy responses while preserving normal vessel development.^153,173–175^ Y1214 phosphorylation activates CDC42, leading to SAPK2/p38 activation, stress fiber formation, and cell migration via NCK and FYN recruitment.^176,177^ Y1212F mutations in mice (*Flk1*^*Y1212F/Y1212F*^;Y1214 in humans) show strain-specific effects, with C57Bl/6 mutants exhibiting partial embryonic lethality and reduced EC proliferation whereas FVB mutants display delayed retinal vascular development and vessel instability, suggesting the essential role of Y1212 in vascular integrity.^178^

Serine phosphorylation at S1183 and S1188 (S1185 and S1190 in humans) in the proline, glutamic acid, serine and threonine (PEST) domain (K1171-K1209) of mouse VEGFR2 has been reported.^179,180^ These phosphorylation recruit β-TRCP1 E3 ligase (Fig. 6b), promoting VEGFR2 ubiquitination and proteasomal degradation. Mutation of these serine residues reduces ubiquitination, whereas phosphomimetic mutations enhance it, underscoring the role of serine phosphorylation in VEGFR2 stability. While tyrosine phosphorylation is well-studied and crucial for VEGFR2 signaling, serine phosphorylation remains less explored and threonine phosphorylation has yet to be identified.

### VEGFR3 signaling

VEGFR3 phosphorylation occurs at several tyrosine residues upon VEGF-C binding with different patterns depending on whether VEGFR3 forms a homodimer or heterodimer with VEGFR2. In VEGFR3 homodimers, five key tyrosine residues are phosphorylated: Y1230, Y1231, Y1265, Y1337, and Y1363 (Fig. 6c). When VEGFR3 forms a heterodimer with VEGFR2, only Y1230, Y1231, and Y1265 are phosphorylated.^131^ Phosphorylation at Y1063 recruits adapter proteins, CRK I/II (Fig. 6c), activating the JNK pathway, which is vital for promoting cell survival. Similarly, phosphorylation at Y1230 and Y1231 plays a key role in recruiting GRB2, triggering the ERK and AKT pathways that drive cell proliferation and migration.^181^ Y1337 has also been identified as a critical phosphorylation site and serves as a docking site for the GRB2-SHC complex (Fig. 6c), which participates in RAS signaling.^182^ In the analysis of families with primary lymphedema, it was found that VEGFR3 proteins carrying mutations such as G857R, R1041P, and L1044P are tyrosine kinase-negative.^183^ Additionally, these mutant VEGFR3 receptors exhibit a longer half-life compared with wild-type receptors. Consequently, they accumulate on the cell surface, potentially contributing to the dominant-negative effects observed in individuals harboring with these mutations.

### NRP signaling

NRPs utilize a conserved binding site structured by the b1 coagulation factor loop, which specifically interacts with the C-terminal arginine motif present in certain VEGF ligands.^184,185^ The interaction of VEGF-A165 with NRP1 is facilitated through specific residues in the b1 domain, where the ligand C-terminal arginine forms a salt bridge with Asp320 in NRP1.^186^ Additional hydrogen bonds between other amino acids stabilize this interaction, allowing for precise isoform-specific binding, which is critical for the selective signaling roles of NRP1 and NRP2.^186^ Structural studies have demonstrated that VEGF-A isoforms bind exclusively to NRP1, while VEGF-C and its related isoforms target NRP2. This distinction enables NRP1 to dominate blood vessel formation and NRP2 to dominate lymphatic vessel development.

NRP1 lacks intrinsic signaling activity. Instead, it relies on its activity to enhance VEGFR2 signaling by recruiting VEGF-A165 and positioning VEGFR2 to amplify downstream pathways.^187^ When VEGF-A165 binds to VEGFR2 with NRP1 assistance, it initiates a cascade of phosphorylation events in VEGFR2. Importantly, NRP1-mediated VEGF-A165/VEGFR2 signaling is also involved in vascular permeability, allowing for better tumor infiltration by blood vessels.^188,189^ NRP2, structurally similar to NRP1, is more selective in lymphangiogenesis, particularly through its interactions with VEGF-C and VEGFR3.^138,190^ NRP2 enhances VEGF-C binding to VEGFR3, facilitating the LEC responses required for lymphangiogenesis. Tumors often induce high expression levels of VEGF-C, which, through the VEGF-C/VEGFR3/NRP2 axis, promotes the proliferation, migration, and survival of LECs.^191,192^ This mechanism is especially relevant in lymphatic metastasis, as lymphatic vessels serve as conduits for tumor cells to reach and colonize distant sites.

### Crosstalk with angiopoietin-TIE receptor

Angiopoietin (Ang) ligands and TIE receptors dynamically interact with the VEGF-VEGFR system to regulate vascular homeostasis and adapt to physiological and pathological changes. Ang1, via TIE2 activation, stabilizes blood vessels, supporting EC survival, vascular barrier integrity, and quiescence. In contrast, Ang2 acts as a context-dependent modulator, often destabilizing vessels under conditions such as inflammation, ischemia, and tumor angiogenesis.^193,194^ By increasing vessel permeability, Ang2 sensitizes ECs to VEGF, enhancing angiogenic sprouting.^195,196^ Notably, VEGF signaling upregulates Ang2, creating a feedback loop where VEGF-induced Ang2 expression counteracts TIE2 stabilization by Ang1.^197^

Additionally, vascular endothelial protein tyrosine phosphatase (VE-PTP) modulates TIE2 and VEGFR2, stabilizing endothelial junctions by dephosphorylating these receptors.^198,199^ Its inhibition enhances TIE2 signaling and strengthens the endothelial barrier, emphasizing the interplay between the VEGF and Ang-TIE pathways in regulating vascular stability. In pathological conditions, such as cancer and retinal diseases, this balance is disrupted. Combined inhibition of VEGF and Ang2, as seen with faricimab, normalizes tumor vessels, reduces vascular permeability, and improves outcomes in diseases such as diabetic macular edema (DME, a complication of diabetes characterized by the accumulation of fluid in the macula due to damaged blood vessels) and AMD.^200–202^ This Ang-TIE-VEGF interplay is critical for vascular stability, and its imbalance contributes to diseases such as cancer.

## Multi-level regulatory mechanisms of VEGF and VEGFR

The VEGF family is tightly regulated at transcriptional and translational levels in response to factors such as hypoxia and metabolic changes. Disruption of this delicate regulation can lead to diseases such as cancer and lymphedema in which abnormal blood vessel growth or lymphatic function becomes a problem.

### Transcriptional and post-transcriptional regulation of VEGF family

The expression of VEGF genes is highly controlled by a complex interplay between transcription factors, signaling pathways, and external stimuli. This network reflects the essential role of VEGFs in physiological processes, as well as in pathological conditions such as cancer.

#### Hypoxia

One of the most significant regulatory mechanisms of VEGF-A transcription is hypoxia, which is mediated by hypoxia-inducible factor 1 (HIF-1).^203^ Under normoxic conditions, HIF-1α is hydroxylated by prolyl hydroxylases, marking it for degradation via the von Hippel-Lindau tumor suppressor protein.^204^ However, under low oxygen conditions, HIF-1α is stabilized, allowing it to dimerize with HIF-1β and bind to the hypoxia response element located in the VEGF-A promoter region.^205^ This binding leads to enhanced transcription of VEGF-A and drives angiogenesis in oxygen-deprived tissues, such as growing tumor. Interestingly, HIF-1-mediated activation is not the only factor involved in VEGF-A regulation under hypoxic conditions. Additional regions upstream of the HIF-1 binding site also contribute to transcriptional activation. For example, the AP1 transcription factor binds to the region between -1168 and -1015 of the promoter,^206^ which is essential for full transcriptional activation under hypoxic conditions, particularly in glioblastoma cells.

#### Hypoxia-independent regulation

Several studies reveal VEGF regulation mechanisms independent of the HIF pathway, highlighting alternative angiogenesis routes. Peroxisome proliferator-activated receptor gamma coactivator 1α (PGC1α) promotes VEGF expression and angiogenesis via estrogen-related receptor α (ERRα) binding to the VEGF gene, identified in muscle cells aiding ischemic recovery.^207^ Oxidative stress also drives VEGF production, as seen in hypertrophied adipocytes releasing VEGF-A120 through the PI3K pathway without hypoxia.^208^ Similarly, cold-induced angiogenesis in adipose tissue, mediated by sympathetic activation and VEGFR2 signaling, further demonstrates hypoxia-independent regulation.^209^ Notably, a nuclear VEGF fragment (N-VEGF) upregulates VEGF-A and hypoxia-related genes independently, offering a novel HIF-bypassing mechanism.^210^

Promoters of VEGF family members exhibit unique regulatory features enabling differential responses to stimuli. VEGF-A and VEGF-B promoters contain Sp1 and AP2 transcription factor binding sites and a CpG island, but VEGF-B uniquely includes Egr-1 sites and lacks AP1 and HIF-1 binding sites, suggesting distinct regulatory pathways.^48,211^ The VEGF-C promoter lacks a TATA box but contains Sp1, AP2, and NF-κB binding sites with a long 5’-untranslated region potentially affecting post-transcriptional regulation.^212^ Additionally, VEGF-D regulation involves a critical direct repeat element in its proximal region, where orphan nuclear receptors HNF-4A and COUP-TF1/TF2 bind coactivators such as CBP and GRIP-1 to enhance expression. The AP1 pathway, particularly c-Fos/c-Jun-dependent, regulates VEGF-D in lung cancer,^213^ with IL-7/IL-7R signaling upregulating VEGF-D via AP1 binding, promoting lymphangiogenesis through enhanced c-Fos/c-Jun heterodimers.

#### N6-methyladenosine modification

N6-methyladenosine (m6A) is a prevalent modification in eukaryotic mRNA that plays a critical role in post-transcriptional regulation. m6A modifications occur at specific adenine residues in a consensus sequence and are dynamically regulated by m6A writers (methyltransferases, such as METTL3 and METTL14), erasers (demethylases, such as FTO and ALKBH5), and readers (binding proteins, such as YTH domain family proteins).^214^ m6A modification in the 5′UTR of VEGF-A mRNA enhances cap-independent translation, driven by METTL3-mediated methylation and YTHDC2/eIF4GI interactions, which promotes VEGF-A expression and tumor progression. Knockdown of METTL3 reduced VEGF-A translation and angiogenesis, demonstrating the therapeutic potential of targeting the m6A/VEGF-A axis in lung cancer.^215^ In addition, Wilms tumor 1-associated protein (WTAP), a key component of the m6A methyltransferase complex, promotes colorectal cancer progression by enhancing VEGF-A through m6A modification mediated by YTHDC1.^216^ Interestingly, YTHDF2, an m6A reader protein, promotes hepatocellular carcinoma (HCC) progression by enhancing ETV5 translation, which upregulates PD-L1 and VEGF-A, driving immune evasion and angiogenesis. Targeting YTHDF2 with siRNA-loaded liposomes effectively reduces tumor growth and restores immune activity, highlighting its therapeutic potential.^217^ m6A modification is a dynamic regulatory mechanism that enhances VEGF-A expression, contributing to tumor progression and angiogenesis in various cancers. Targeting key components of the m6A machinery, such as METTL3, WTAP, and YTHDF2, offers promising therapeutic strategies for inhibiting VEGF-A-driven oncogenic pathways.

#### MicroRNAs and non-coding RNAs

MicroRNAs (miRNAs) are small, evolutionarily conserved non-coding RNAs, approximately twenty-two nucleotides in length, first discovered in *C. elegans*.^218^ Typically, miRNAs act as negative regulators by binding to complementary sequences in the 3′-UTR of mRNAs, promoting mRNA degradation or inhibiting translation.^219^ This post-transcriptional regulation is essential for controlling homeostatic and developmental events.

miR-126 is a key regulator of VEGF signaling,^220^ which elevates angiogenesis by suppressing negative regulators such as SPRED1 and PIK3R2, affecting the MAPK and PI3K pathways.^221^ miR-126 downregulation elevates VEGF-A expression, driving tumor angiogenesis. Epigenetic silencing of miR-126 increases VEGF-A levels, enhancing neovascularization and metastasis.^222^ Conversely, miR-16 directly targets VEGF-A in cancers such as multiple myeloma and lung cancer, reducing tumor growth and angiogenesis.^223,224^ Similarly, miR-205 is a key regulator in various cancers, affecting angiogenesis, metastasis, and chemoresistance. In breast cancer, miR-205 enhances chemosensitivity by targeting VEGF-A and FGF2, reducing resistance to drugs, such as doxorubicin and paclitaxel, and promoting apoptosis through the PI3K/AKT pathway.^225^ In HCC, miR-205 inhibits tumor growth and metastasis by directly targeting VEGF-A, suggesting its potential as a therapeutic target.^226^ Additionally, in breast cancer-associated fibroblasts, miR-205 mediates VEGF-independent angiogenesis through the YAP1-IL-11/IL-15-STAT3 axis, providing new strategies to combat resistance to anti-VEGF treatments.^227^ These findings underscore the dual role of miRNAs in regulating VEGF-A expression and angiogenesis, either promoting or inhibiting tumor growth and metastasis. The activity of specific miRNAs, such as miR-126 and miR-205, to modulate key pathways associated with tumor progression and angiogenesis highlights their significance in disease mechanisms and their potential as therapeutic targets.

Long non-coding RNAs (lncRNAs), RNA molecules over 200 nucleotides long, regulate gene expression without encoding proteins.^228,229^ They function through various mechanisms such as chromatin remodeling or acting as decoys for proteins or miRNAs. Maternally expressed gene 3 (MEG3), a key lncRNA, regulates angiogenesis and vascular function by exerting anti-proliferative and pro-apoptotic effects, partly through p53 accumulation.^229–231^ In DR, MEG3 downregulation exacerbates microvascular damage by increasing VEGF and inflammatory cytokines.^232,233^ MEG3 also regulates VEGFR2 expression in ECs, and its reduction impairs VEGF-driven angiogenesis.^234^ Overexpression of MEG3 can mitigate high glucose-induced VEGF and TGFβ1 levels, suggesting therapeutic potential for pathological angiogenesis.^233^ In contrast, the lncRNA MALAT1 plays a pro-angiogenic role. In DR, MALAT1 supported EC growth and migration via the YAP1/miR-200b-3p/VEGF-A pathway.^235^ In retinopathy of prematurity (ROP, eye disorder of abnormal retinal blood vessel development in premature infants), MALAT1 inhibition with siRNA reduced retinal neovascularization and lowered VEGF and inflammatory markers, highlighting its role in abnormal retinal vessel development.^236^ Together, MEG3 and MALAT1 illustrate the diverse and opposing roles of lncRNAs in angiogenesis and vascular pathology.

## Post-translational regulation

Post-translational modifications enhance protein diversity and help cells maintain homeostasis by altering the protein structure, stability, and interactions.

### Glycosylation

Glycosylation of VEGF and VEGFR family members plays a subtle yet key role in their stability, secretion, and interactions, although it is not always essential for core functions, such as receptor binding. For VEGF-A, glycosylation at Asn74 (N74) is not essential for receptor binding or EC proliferation,^237^ although it enhances protein stability and binding to GAGs. Similarly, VEGF-B186 is glycosylated in its C-terminal region, increasing its molecular weight and solubility compared with the non-glycosylated VEGF-B167, facilitating its secretion and extracellular stability.^14^ N-glycosylation is critical for VEGF-D, as glycosylation at N155 and N185 ensures proper folding, solubility, and secretion.^238^ Similarly, for VEGFR2, glycosylation at N247 regulates receptor activation, specifically, removing sialylated N-glycans at this site increases ligand-induced activation, which, in turn, regulates receptor dimerization and downstream signaling.^239^ Moreover, glycosylation and sialylation of VEGFR2 regulate its stability, trafficking, and receptor-ligand interactions, underscoring their critical role in regulating VEGF/VEGFR signaling and function.

### SUMOylation

SUMOylation is a reversible process in which small ubiquitin-like modifier (SUMO) proteins are attached to target proteins, and regulate key functions, such as DNA repair.^240,241^ This modification involves a series of enzymes (E1, E2, and E3) that attach SUMO to specific lysine residues, while SUMO-specific proteases (SENPs) remove it, allowing for dynamic control of protein activity. SUMOylation of Lys1270 of VEGFR2 leads to its retention in the Golgi, reducing its expression on the cell surface and impairing VEGFR2 signaling.^242^ SENP1, a SUMO-specific protease, removes this modification, allowing VEGFR2 to reach the cell membrane and activate angiogenesis.^242,243^ In SENP1-deficient cells, VEGFR2 remains hyper-SUMOylated and trapped in the Golgi, leading to impaired angiogenesis. Moreover, SENP6 deSUMOylates VEGFR2, promoting its transport from the Golgi to the cell membrane and enhancing VEGFR2-mediated angiogenesis.^244^ This process is particularly important in conditions such as diabetic microangiopathy, where altered SUMOylation dynamics can affect pathological angiogenesis.^244^ Similarly, SUMOylation at Lys1270 inhibits VEGFR2 activity by reducing its phosphorylation and downstream signaling pathways, such as the AKT and ERK pathways. This modification also impairs cell proliferation and migration while promoting apoptosis in non-small cell lung carcinoma cells. Conversely, deSUMOylation of VEGFR2 by enzymes such as SENP1 can enhance VEGFR2 activity and restore angiogenic signaling.^245^

### Ubiquitination

VEGFR2 ubiquitination is a critical post-translational modification that regulates its signaling, stability, and degradation, and directly affects angiogenesis. Among the key regulators, various E3 ubiquitin ligases, such as c-CBL, β-TRCP, and RNF121, activate VEGFR2 with ubiquitin, marking it for degradation through the endosomal-lysosomal system. This process is particularly important because ligand-dependent ubiquitination is necessary for regulating receptor expression on the membrane and preventing overstimulation of VEGF-A signaling.^246,247^ In addition, the CUL3-SPOP-DAXX axis has been identified as a key regulator of VEGFR2 expression.^248,249^ This complex facilitates ubiquitination of VEGFR2, reducing its expression and subsequently limiting angiogenic signaling. Moreover, E2 ubiquitin-conjugating enzymes such as UBE2D1 and UBE2D2 play crucial roles in modulating VEGFR2 ubiquitination. Notably, their depletion leads to increased VEGFR2 levels, enhancing VEGF-A-stimulated signaling pathways, such as MAPK, AKT, and PLCγ1, and promoting EC migration and tubulogenesis.^250^ Thus, the balance of ubiquitination, recycling, and degradation orchestrates the intensity and duration of VEGFR2 signaling in physiological and pathological angiogenesis. Conversely, USP8, a deubiquitinating enzyme, removes ubiquitin chains from VEGFR2, facilitating its recycling from early endosomes back to the membrane and preventing its degradation in the lysosome.^251^ However, when USP8 is depleted, VEGFR2 accumulates in early endosomes, leading to the generation of a novel 120 kDa proteolytic fragment. As a result, this accumulation also impairs VEGF-A-induced signaling, particularly through the AKT and ERK pathways.

## Regulatory mechanisms of VEGFR activity

VEGFR activity is tightly regulated by various molecular processes that maintain vascular homeostasis and modulate the angiogenic response. These regulatory mechanisms include both positive and negative modulators, which fine-tune VEGFRs signaling to prevent excessive or insufficient angiogenesis.

### Protein phosphatase

**Fig. 7:**
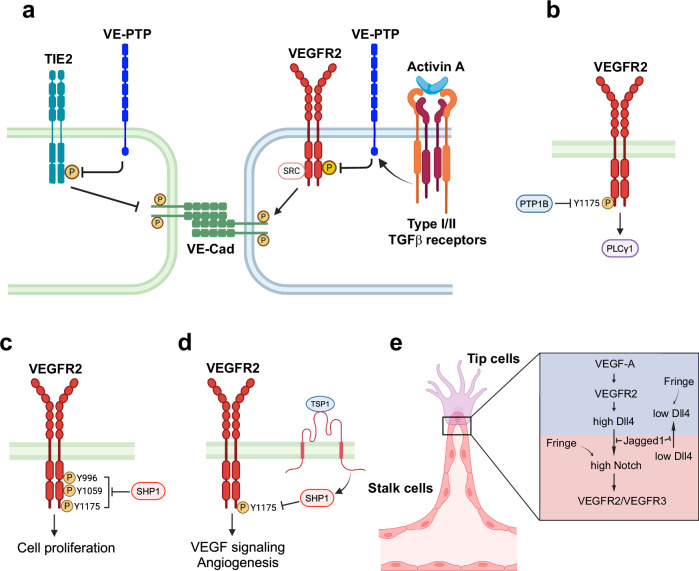
Negative regulation and fine-tuning of VEGFR2-mediated signaling pathways. **a** VE-PTP regulates TIE2 and VEGFR2 dephosphorylation to balance angiogenic signaling and support vessel integrity. Activin A enhances VE-PTP expression and reduces VEGF-induced permeability, which may help control excessive vessel leakage in conditions such as diabetic retinopathy. **b** PTP1B regulates VEGFR2 signaling by binding to its cytoplasmic domain and dephosphorylating it, thereby acting as a negative regulator. **c** SHP1 acts as a negative regulator of VEGFR2 by dephosphorylating key tyrosine residues (Y996, Y1059, and Y1175) essential for endothelial cell proliferation. **d** TSP-1 modulates VEGFR2 activity by binding to CD36 and recruiting SHP1, which dephosphorylates VEGFR2 at Y1175. This action suppresses VEGF signaling and inhibits angiogenesis. **e** Notch signaling coordinates angiogenesis by balancing the roles of tip and stalk cells. Dll4 on tip cells activates Notch in stalk cells, reducing VEGFR expression and VEGF sensitivity to stabilize newly formed vessels. Fringe, a glycosyltransferase in stalk cells, enhances Dll4-Notch signaling, further reducing VEGFR levels and reinforcing stalk cell quiescence. Meanwhile, Jagged1 counteracts Dll4 by modulating Notch in stalk cells, allowing some cells to remain responsive to VEGF, supporting sprouting. This interplay ensures selective sprouting while maintaining vessel stability. Notably, the Notch-VEGFR3 pathway can also promote angiogenesis independently of VEGF-A/VEGFR2. VE-PTP vascular endothelial protein tyrosine phosphatase, VEGFR vascular endothelial growth factor receptor, PTP1B protein tyrosine phosphatase 1B, SHP-1 Src homology 2 domain-containing phosphatase-1, TSP-1 Thrombospondin-1. Created in BioRendender.com

VE-PTP, a receptor-type phosphatase, is crucial for regulating endothelial junctions and blood vessel development.^252,253^ VE-PTP is highly expressed in ECs, especially during vascular development, VE-PTP interacts with TIE2 (Fig. 7a), dephosphorylating it to regulate Ang/TIE2 signaling.^254^ It also negatively regulates VEGFR2 phosphorylation at endothelial junctions, controlling VEGFR2 activity and preventing excessive angiogenic signaling.^255^ VE-PTP ensures proper EC polarity and lumen formation with its deficiency leading to increased VEGFR2 phosphorylation, abnormal vessel sprouting and endothelial dysfunction.^256,257^ Recent studies showed that activin A, a member of the TGFβ family, reduces VEGF-induced vascular permeability by increasing VE-PTP expression which dephosphorylates VEGFR2 and dampens VEGF signaling,^258^ suggesting therapeutic potential for conditions such as DR (Fig. 7a).

PTP1B, another phosphatase, binds to the cytoplasmic domain of VEGFR2 and directly dephosphorylates it (Fig. 7b), acting as a negative regulator.^259^ Overexpression of PTP1B reduces VEGF-induced VEGFR2 autophosphorylation, inhibiting downstream signaling ERK signaling and EC proliferation. PTP1B also dephosphorylates VEGFR2 at Y1175 site during endocytic trafficking, suppressing VEGF signaling and the PLCγ1-MAPK pathway essential for arterial development.^260^ Similarly, SHP1 negatively regulates VEGFR2 by dephosphorylating key tyrosine residues (Y996, Y1059, Y1175) (Fig. 7c) upon VEGF stimulation via a c-SRC-dependent mechanism.^261^ In addition, thrombospondin-1 (TSP-1) enhances this regulation by binding to CD36 and recruiting SHP1 to dephosphorylate VEGFR2 at Y1175 (Fig. 7d), further suppressing VEGF signaling and angiogenesis.^262^ While this study focuses on tyrosine phosphatases, serine/threonine phosphatases also play distinct roles in VEGFR2 regulation and downstream signaling.

### Negative feedback regulator

Negative feedback regulation maintains homeostasis by reducing or turning off a signaling pathway once the desired response is achieved.^263^ Key regulators such as Sprouty, suppressor of cytokine signaling (SOCS), and ErbB receptor feedback inhibitor-1 (ERRFI1) inhibit excessive signaling by targeting pathways such as RTKs, JAK/STAT, and RAS/MAPK pathways, ensuring tight control over signaling cascades.^264,265^

Vasohibin (VASH), identified as a VEGF-inducible gene in ECs, acts as a negative regulator of angiogenesis.^266,267^ VASH inhibits EC migration, proliferation, and network formation, specifically targeting ECs without affecting other cell types.^266,268^ Its expression is downregulated by hypoxia and inflammatory cytokines, such as TNF-α, potentially impairing its anti-angiogenic activity in conditions such as cancer. VASH has two isoforms: VASH1 and VASH2. VASH1, with two variants (VASH1A and VASH1B), primarily functions as an anti-angiogenic factor.^269^ VASH1A normalizes tumor vessels, improving perfusion and chemotherapy efficacy, while VASH1B induces autophagic cell death in ECs, leading to vascular pruning and increased hypoxia.^270^ In contrast, VASH2 promotes angiogenesis and is highly expressed in cancers, supporting tumor growth and metastasis by facilitating new blood vessel formation.^271–273^ This dual role of VASH, acting as both an inhibitor and a promoter of angiogenesis, highlights its dynamic function in tumor development and therapeutic outcomes.

The VEGF/VEGFR and Notch/Dll4 pathways function together in a finely tuned negative feedback loop.^274,275^ Indeed, Notch signaling is essential for angiogenesis, coordinating blood vessel formation.^276,277^ When Dll4 on endothelial tip cells binds to Notch receptors on neighboring stalk cells, it triggers the release of Notch intracellular domain (NICD), which moves to the nucleus to affect gene expression. This Notch/Dll4 signaling balances tip and stalk cell roles during vessel sprouting and acts closely with VEGF signaling to maintain blood vessel formation (Fig. 7e). Tip cells, which lead to new vessel sprouting, are highly affected by VEGF-A/VEGFR2 activation, which triggers strong ERK signaling and upregulates tip cell-specific genes such as Dll4.^278–280^ In turn, Dll4 interacts with Notch receptors in nearby stalk cells, reducing their expression of VEGFR2 and VEGFR3 and making them less responsive to VEGF.^281,282^ As a result, these cells adopt the role of stabilizing newly formed vessels rather than participating in further sprouting. This feedback loop ensures that stalk cells stabilize new vessels rather than participating in further sprouting, maintaining a balance between sprouting and stabilization.^281,282^ This tightly regulated interaction is vital for proper vascular development, as it ensures that sprouting occurs in a controlled manner.

In contrast, Jagged1, another Notch ligand, promotes angiogenesis by antagonizing Dll4-Notch signaling (Fig. 7e), especially in cells expressing the Fringe glycosyltransferase.^283^ Fringe enhances Dll4-Notch signaling while diminishing the Jagged1 effect, allowing Jagged1 to support tip cell formation and sprouting. This interplay between Dll4 and Jagged1 finely balances angiogenesis. Interestingly, Notch signaling can drive angiogenesis independently of VEGF-A and VEGFR2 by upregulating VEGFR3, enabling vessel sprouting even when VEGFR2 is inhibited.^284^ Blocking Notch signaling increased sprouting and vessel density in the absence of VEGFR2, revealing a VEGF-A-independent mechanism. This challenges the traditional reliance on VEGF-A/VEGFR2 in angiogenesis and suggests that targeting Notch and VEGFR3 could offer new strategies, particularly for tumors resistant to anti-VEGF treatment. Overall, this complex interplay between Notch, Dll4, Jagged1, VEGF, and VEGFR signaling pathways forms a multilayered feedback system that precisely controls angiogenic sprouting and vessel stability, highlighting a sophisticated regulatory network essential for balanced vascular growth.

## Physiological roles of VEGF/VEGFR signaling

In multicellular animals, the slow efficiency of oxygen and nutrient diffusion limits their reach to short distances.^285^ To address the high demand for oxygen and nutrients driven by body size expansion during evolution, the development of an internal transport and exchange system became essential for substance delivery and waste removal. Invertebrates evolved circulatory systems - both open and closed - with vessels lined by ECM.^286^ Vertebrates, however, developed a closed circulatory system featuring vessels with a true endothelial lining.^287^ This endothelial lining, composed of tightly interconnected ECs anchored to a basement membrane (BM) with basoapical polarity, represents an evolutionary innovation. Beyond forming a functional vasculature for oxygen and nutrient delivery, ECs interact with perivascular cells to regulate blood flow, modulate immune cell trafficking, and produce signaling molecules to maintain tissue homeostasis.

### Angiogenesis

Angiogenesis has been recognized since ancient times, with early descriptions by the Greek physician Galen, who compared embryonic growth along umbilical veins to plant growth.^288,289^ While its morphological features were detailed in the 1960s,^290,291^ the molecular mechanisms were not elucidated until the 1970s by Judah Folkman and others.^292^

**Fig. 8:**
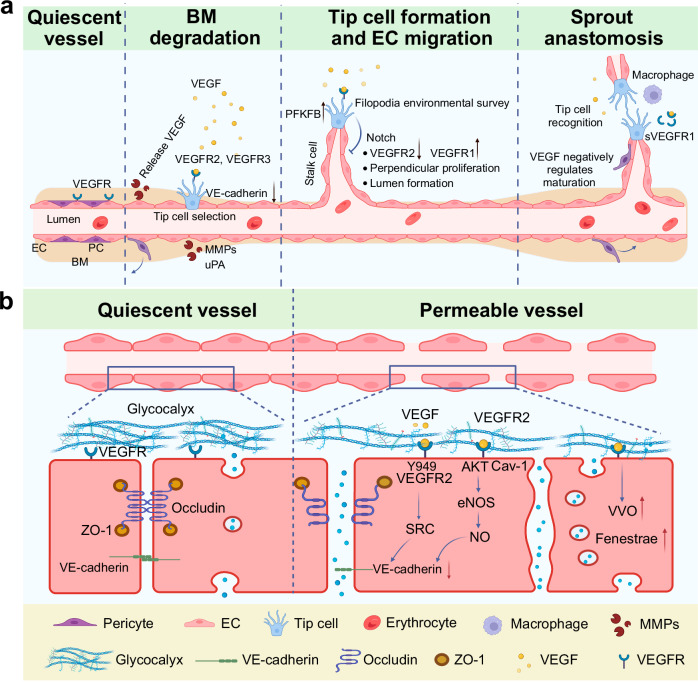
VEGF/VEGFR signaling controls angiogenesis and vascular permeability. **a** Role of VEGF/VEGFR signaling pathway in angiogenesis. (1) The vascular stability of quiescent microvessels covered by pericytes and basement membrane (BM) is maintained by non-VEGF/VEGFR signaling pathways such as Ang/TIE signaling. (2) Upon VEGF ligand stimulation, the BM degrades and releases bio-unavailable VEGF ligands. Endothelial cells (ECs) lose structural support and key adhesion molecules such as VE-cadherin and undergo tip cell selection. (3) VEGF signaling strongly induces the tip cell phenotype and metabolic characteristics, providing guidance cue for tip cells, whereas VEGFR2 and VEGFR3 are highly expressed in tip cells. Tip cells inhibit VEGFR2 and VEGFR3 and increase VEGFR1 and sVEGFR1 in stalk cells. VEGF supports perpendicular proliferation of stalk cells and lumen formation. (4) Tip cells recognize each other and induce sprout anastomosis. sVEGFR1, possibly produced by macrophages, regulates this process. VEGF negatively regulates vessel maturation. **b** Role of VEGF/VEGFR signaling in vascular permeability. The vascular permeability of quiescent microvessels is maintained by pores in ECs, glycocalyx, and EC junctions. VEGFR2 Y949 phosphosite in mice downregulates VE-cadherin via SRC. VEGFR2 also promotes nitric oxide (NO) production to reduce the expression of adhesion molecules required for paracellular permeability. VEGF has also been shown to stimulate vesicovacuolar organelles (VVO) and fenestrae formation for transcellular permeability. However, whether the glycocalyx can be cleaved by VEGF stimulation is not fully understood. Created in BioRendender.com

Angiogenesis begins (Fig. 8a) with (1) the degradation of BM and loss of pericyte coverage in response to angiogenic stimuli. (2) ECs then migrate toward the stimulus with stalk cells aligning under the control of tip cells. (3) tip cells survey the microenvironment and connect to sprouts. Following this, (4) vessel maturation occurs, involving new BM formation and coverage by perivascular cell types, (5) blood flow is established, supporting the survival of new vessels.^293,294^ Angiogenesis is tightly regulated by a balance of pro-angiogenic and anti-angiogenic factors. Among these, VEGF/VEGFR signaling is the best-defined key stimulator, demonstrated in models such as chicken embryos,^11^ and primate,^295^ and plays a critical role in every step of angiogenesis.

#### VEGF in BM degradation

During BM degradation step, ECs lose structural support and tissues undergo decompartmentalization, enabling sprouting. The BM, composed of collagen,^296^ laminin,^297^ and elastin,^298^ and inter-EC adhesion molecules such as VE-cad,^299^ claudin,^300^ and junctional adhesion molecules,^301^ separates blood from underlying tissue. BM degradation, mediated by the proteolytic process of urokinase plasminogen activator (uPA) and matrix metalloproteinases (MMPs), facilitates angiogenesis.^302^ ECM-degrading enzymes regulate VEGF signaling through:

1. releasing bio-unavailable ligands: due to the varying heparin-binding and heparan sulfate-binding capacities of VEGF isoforms, some VEGF ligands are sequestered in the ECM. These bio-unavailable VEGF ligands are released following proteoglycan degradation, promoting angiogenesis^303^;
2. directly cleaving matrix-bound VEGF isoforms to modulate their angiogenic properties;^304^ and
3. stimulating VEGF expression via membrane-type MMP and SRC tyrosine kinases.^305,306^ Following degradation, ECM fragments such as endorepellin,^307^ endostatin,^308^ thrombospondin-1,^309^ and tumstatin,^310^ often act as endogenous inhibitors, balancing pro-angiogenic and anti-angiogenic signals.

Accompanying BM degradation, VEGF also disrupts inter-EC adhesion in quiescent vessels by:

1. adherens junction dissociation: VEGF induces rapid dissociation of the EC-specific tyrosine phosphatase VE-PTP from VE-cad (Fig. 8b), thereby disrupting the endothelial barrier;^311^
2. endocytosis of adhesion molecules: VEGF stimulation promotes endocytosis of VE-cad, enabling EC migration and proliferation;^312^ and
3. regulating adherens junctions to control tight junctions: VE-cad promotes intracellular signaling that upregulates claudin-5, a major component of tight junction component, ensuring coordinated regulation of endothelial permeability.^313^

Thus, VEGF downregulates both junction types and promotes angiogenesis. Conversely, adherens junction proteins, including VE-cad and PECAM1 (also known as CD31) can be phosphorylated by mechanical forces, activating VEGFR-related signaling.^314,315^ This integrates mechanical and chemical stimuli in ECs, driving angiogenesis.^316^

#### VEGF in tip cell formation and EC migration

Once the barrier is breached, a small subset of ECs undergoes an angiogenic switch, enabling migration and proliferation. At this stage, tip cells emerge from this EC subpopulation, becoming the leading cells of sprouting vessels (Fig. 8a). Morphologically distinct, tip cells function as sensors, using dynamic filopodia to survey the environment for guidance cues and direct migration. This process resembles the insect tracheal system, where specialized cells guide migration and determine cell fate.^317^ Interestingly, although tip cells were observed in the 1970s,^318^ their molecular understanding emerged in the 2000s.^293^

VEGF signaling strongly drives the tip cell phenotype. During retinal development, tip cells exhibit high VEGFR2 expression compared with stalk or quiescent ECs.^319^ VEGF ligands guide tip cell migration and are essential for tip cell filopodia formation, extension, and orientation.^319^ In addition to VEGF/VEGFR2 signaling, VEGFR3 is highly expressed in tip cells and sustains angiogenesis even when VEGFR2 is inhibited.^320^ VEGFR2/3 heterodimers often found on tip cell filopodia,^132^ likely mediate responses to VEGF-A and VEGF-C in vivo. Beyond regulating tip cell behavior, VEGF also affects its metabolic phenotype. While ECs in perfused vessels rely on oxygen, VEGF-driven tip cells depend on glycolysis, mediated by 6-phosphofructo-2-kinase/fructose-2,6-bisphosphatase (PFKFB).^321,322^

Uncontrolled tip cell formation results in dysfunctional vessels, so tip cells must regulate neighboring ECs to maintain the stalk cell phenotype. In zebrafish, VEGFR1 is highly expressed in stalk cells, and its loss leads to excessive tip cells and hyperbranching, indicating that VEGFR1 negatively regulates the tip cell phenotype.^323^ Furthermore, through Notch signaling, tip cells suppress VEGFR2 and VEGFR3 expression whereas increasing VEGFR1 and sVEGFR1 in stalk cells, reducing their sensitivity to VEGF and maintain the stalk phenotype.^277,324^ Tip cells also produce various immune and angiogenic modulators that facilitate tip-stalk cell communication.^325^ Importantly, tip and stalk cells are not fixed phenotypes. Therefore, continuous communication is essential to sustain their distinct roles during angiogenesis.^326^

Stalk cells follow tip cells, proliferating perpendicular to their guidance to elongate vessels and form lumens for tubular morphogenesis. Disrupted VEGF signaling misorients stalk cell division, causing vessel dysmorphogenesis, indicating a flow-independent regulatory mechanism.^327^ During lumen formation, cell-cell contact is crucial for EC shape changes. In mouse aortic development, VEGF recruits myosin to apical cell surfaces at EC contact sites, driving morphological alterations.^328^

#### VEGF in sprout anastomosis

The mechanism of tip cell fusion during sprout anastomosis remains unclear but appears to involve tip cell recognition, EC polarization, and apical membrane fusion. VE-cad and filopodia are essential for this process.^329,330^ Interestingly, the spatial distribution of VEGFR1 regulates anastomosis, as in vitro studies show that tip cells make transient contacts before stable vessel connection, while VEGFR1 reduces contact frequency and alters vessel connection site.^331^ Perivascular cells may act as chaperones^332^ and resident macrophages, which secrete sVEGFR1 via a non-canonical pathway,^333^ could play a regulatory role in sprout anastomosis.

#### VEGF in vessel maturation

After sprouting, vessels undergo remodeling and maturation, a critical step for establishing a functional vascular network.^334^ Blood flow delivers oxygen and mechanical forces that shape vascular remodeling.^335,336^ As vessels mature, ECs form a new BM to stabilize and pattern the vasculature.^337^ Perivascular cells, including pericytes, are recruited and stabilize vessels through non-VEGF signals such as PDGF, TGFβ, Ang1, and Notch.^338,339^ Notably, VEGF signaling inhibits vessel maturation, highlighting its role in early angiogenesis rather than stabilization.^142^

#### Interplay between VEGF and other pro-angiogenic factors

Several angiogenic factors, including FGFs and Ang, stimulate angiogenesis under physiological conditions, differing from VEGF pathways.^340,341^ For example, FGFs are widely expressed, promoting EC proliferation,^342^ whereas VEGF is mainly EC-specific, exerting diverse regulatory effects. During development, FGF1 and FGF2 are less critical,^343^ whereas TIE2, expressed in ECs, interacts with Ang1 and Ang2. Ang1 stabilizes quiescent blood vessels,^344^ whereas Ang2 modulates the Ang1/TIE2 axis, sensitizing vessels to VEGF and other factors.^341^ Ang1 and VEGF complement in each other in early vascular development, with VEGF initiating vessel formation and Ang1 supporting subsequent maturation.^345^ Ang2 is required for post-natal angiogenesis.^346^ These factors, along with VEGF, synergistically orchestrate angiogenesis to meet tissue-specific vascular needs.^347,348^

VEGF signaling is central to every step of angiogenesis (Fig. 8a), yet its exact role in processes such as sprout anastomosis remain unclear due to EC heterogeneity and dynamic phenotypic changes. The involvement of other cell types also requires further investigation. Recent single-cell studies may provide deeper insights.^349^ While the molecular mechanisms of VEGF signaling in angiogenesis are well understood, angiogenesis relies on precise spatiotemporal regulation to ensure proper vascular development. A comprehensive understanding of these dynamic processes may open new avenues for therapeutic strategies targeting pathological angiogenesis.

### Vasculogenesis and other types of vascular formation

Vasculogenesis involves the de novo formation of blood vessels from endothelial progenitor cells (EPC), occurring during early embryonic development or rapid vascularization of avascular tissues.^350,351^ Endothelial and hematopoietic progenitor cells arise from the embryonic mesoderm, yolk sac, allantois, and placenta, migrating to blood islands where they differentiate into angioblasts (forming ECs) and hematopoietic precursors (forming blood cells).^352,353^ Differentiated ECs polarize and form a vascular lumen, through intracellular vesicle fusion or slit formation,^354^ eventually forming major vessels such as aorta or middle cerebral artery after BM deposition.^355^

Morphogens including Wnt, Hedgehog, TGFβ, and FGFs, collectively regulate blood island formation, angioblast induction, and yolk sac vasculogenesis.^356^ Additionally, VEGF, expressed in the extra-embryonic endoderm and mesoderm,^357^ is crucial for vasculogenesis; for instance, embryos lacking VEGF, VEGFR2, or VEGFR3 exhibit severe defect (Table 1), including failed blood island formation, aortic abnormalities, or large vessel defects,^358–361^ Moreover, VEGFR1 inhibits EC overgrowth and thereby tightly regulates VEGF signaling during vasculogenesis.^362^

**Table 1:** Phenotype analysis of VEGF and VEGFR mouse models

Mouse model	Targeted gene	Targeting strategy	Phenotype	Reference
*Vegfa*^-/-^	*Vegfa*	Exon 3 replaced with neomycin resistance cassette	Null mutation, embryonic lethality (E8.5–9.5), severe vascular defects	^358,359^
*Vegfa120/120*	*Vegfa120 (*Soluble isoform only*)*	Exons 6 and 7 replaced with neomycin resistance cassette	Vascular defects, impaired vessel patterning, cardiac abnormalities, early postnatal lethality	^700^
*Vegfa164/164*	*Vegfa164*	Replacement of exons 4, 5, 7, 8	Normal vascular development, sufficient for vascular growth and remodeling	
*Vegfa188/188*	*Vegfa188*	Replacement of exons 4 through 8	Normal venular growth, arteriolar defects, underdeveloped arterioles	
*Vegfa*^*loxP/loxP*^ x *VE-Cad-Cre*	*Vegfa* (ECs)	Exon 3 flanked by loxP sites, VE-Cadherin promoter drives Cre	Vascular degeneration, EC apoptosis, hemorrhage, cardiac dysfunction, early death	^416^
*Vegfa*^*loxP/loxP*^ x *Mlc2v-Cre*	*Vegfa* (Cardiac muscle)	Exon 3 flanked by loxP sites, Mlc2v promoter drives Cre	Thin, dilated ventricles, hypovascularization, reduced cardiac function	^454^
*Vegfa*^*loxP/loxP*^ x *VMD2-Cre*	*Vegfa* (RPE cells)	Exon 3 flanked by loxP sites, VMD2 promoter drives Cre	Loss of choriocapillaris, rapid cone photoreceptor dysfunction, severe vision loss	^535^
*Vegfb*^*-/-*^	*Vegfb*	Exons 3 to 7 replaced with promoter β-geo cassette	Smaller heart, reduced coronary function, impaired recovery from ischemia, otherwise healthy	^391^
*Vegfb*^*loxP/loxP*^ x *Adipoq-Cre*	*Vegfb* (Adipocytes)	LoxP sites in intron 1 and 6, Adipoq promoter drives Cre	Reduced plasma non-esterified fatty acids, improved insulin sensitivity, reduced ischemic stroke damage	^701^
*Vegfb*^*loxP/loxP*^ x *RIP-Cre*	*Vegfb* (β-cells)	LoxP sites between exons 1, 2 and 6, 7, RIP promoter drives Cre	Increased insulin gene expression, enhanced insulin secretion, no significant effect on glucose tolerance	^702^
*Vegfb*^*loxP/loxP*^ x *Cd4-Cre*	*Vegfb* (T cells)	LoxP sites around exons 2-6, CD4 promoter drives Cre	Increased CD4 + /CD8 + T cell apoptosis, impaired mitochondrial function, reduced T-cell survival and memory, alleviated autoimmune disease symptoms	^703^
*Vegfc*^*-/-*^	*Vegfc*	Exon 1 replaced with lacZ-neo cassette, removing translation start	Severe lymphatic defects, absence of lymphatic vessels, prenatal lethality by E12.5	^375^
*Vegfc*^*loxP/loxP*^ x *Rosa-CreER*^*T2*^	*Vegfc*	LoxP sites around exon 3, tamoxifen-inducible Cre with Rosa-CreERT2	Impaired lymphangiogenesis, compromised lymphatic repair mechanisms	^704^
*Vegfd*^*-/-*^	*Vegfd*	Signal sequence and first/second coding exons replaced with lacZ-neo	Viable, normal lymphatic development, no significant developmental abnormalities	^376^
*Plgf*^*-/-*^	*Plgf*	Exons 3 to 6 deleted	Normal embryonic development, impaired pathological angiogenesis in ischemia, inflammation, wound healing, tumors	^87^
*Vegfr-1*^*-/-*^ *(Flt1*^*-/-*^*)*	*Vegfr1*	Exon encoding signal peptide replaced with lacZ-neo, creating null allele	Severe vascular defects, embryonic lethality (E8.5), disorganized vasculature in yolk sac	^362^
*Vegfr1*^*loxP/*loxP^ x *Rosa26-CreER*^*T2*^	*Vegfr1*	LoxP sites around exon 3, tamoxifen-inducible Cre	Enhanced angiogenesis, increased retinal vessel density and branching, sensitivity to VEGF-A-induced permeability	^705^
*Vegfr1(Flt1)-tk-KO*	*Vegfr1* (deletion of *Flt1* tyrosine kinase)	Exon 17 replaced with neomycin cassette, removing tyrosine kinase domain	Normal vascular development, reduced VEGF-induced macrophage migration, tyrosine kinase domain required for macrophage response	^706^
*Vegfr2*^*-/-*^ *(flk1*^*-/-*^*)*	*Vegfr2*	First coding exon replaced with lacZ-neo	Embryonic lethality (E8.5–9.5), severe vasculogenesis defects, absence of organized blood vessels	^360^
*Vegfr2*^*loxP/loxP*^ x *VE-Cad-Cre*	*Vegfr2* (ECs)	LoxP sites around critical exon, VE-cadherin promoter drives Cre	Impaired angiogenesis, reduced endothelial sprouting, decreased retinal vessel density	^284^
*Vegfr2*^*loxP/loxP*^ x *Nestin-Cre*	*Vegfr2* (Neural progenitors)	LoxP sites flanking essential exons, Nestin promoter drives Cre	Impaired cortical and hippocampal vascularization, reduced brain vascular density	^707^
*Vegfr2*^*loxP/loxP*^ x *Lyve1-Cre*	*Vegfr2* (LECs)	LoxP sites around essential exons, Lyve-1 promoter drives Cre	Lymphatic hypoplasia, reduced lymphatic vessel density in skin, embryonic lethality (E14.5)	^708^
*Vegfr3*^*-/-*^	*Vegfr3*	Translation start codon replaced with LacZ cassette	Defective cardiovascular development, severe vascular malformations, embryonic lethality by E9.5	^361^
*Vegfr3*^*ΔLBD/ΔLBD*^*, Vegfr3*^*tyrosine kinase mutant*^	*Vegfr3* (LBD, tyrosine kinase domain)	Deletion or point mutation of LBD and kinase domains	LBD deletion: severe lymphatic defects, impaired lymphangiogenesis; Tyrosine kinase mutant: impaired lymphatic development but normal angiogenesis	^377^

*EC* endothelial cell, *Mlc2v* myosin light chain-2, *VMD2* vitelliform macular dystrophy, *RPE* retinal pigment epithelium, *tk* tyrosine kinase, *LEC* lymphatic endothelial cells, *LBD* ligand binding domain

Intussusceptive angiogenesis involves vessels splitting to form new vessels, observed in developing organs and pathological vascular remodeling.^363,364^ During this process, transluminal tissue pillars develop and fuse, creating new vascular structures. While VEGF and Notch signaling are implicated in intussusceptive angiogenesis,^365,366^ the detailed molecular mechanisms remain unclear. Alternatively, another mode of vascular formation involves the segregation of cells from a common precursor vessel into discrete arterial and venous vessels, as seen in zebrafish embryo development. Specifically, VEGF-induced Ephb2a directs angioblast migration, determining vessel identity.^367^ Compared with angiogenesis, vasculogenesis and other types of vascular formation are less studied with most descriptive studies, offering limited insights into molecular mechanisms. This is partly due to the transient nature of these processes and their overlapping occurrence in vivo, which complicates understanding. To address this, models that can specifically induce distinct forms of vascular formation are needed to clarify whether and how VEGF signaling affects these processes.

### Lymphangiogenesis

Approximately 400 years ago, lymphatic vessels were first described by Gaspare Aselli in Italy and their route was correctly identified by Jean Pecquet in France.^368^ Functioning as a parallel circulatory system to blood vessels, lymphatic vessels maintain tissue homeostasis by draining interstitial fluid and transporting antigens and immune cells to lymph nodes for immune surveillance. During early development, lymphatic vessels originate from the cardinal,^369^ as demonstrated by genetic tracing of Prox1 cells in mice.^370^

Since the 1990s, lymphangiogenic factors have been identified with the VEGF-C/D-VEGFR3 axis emerging as the most critical signaling pathway regulating lymphangiogenesis.^371,372^ Notably, VEGF-C transgenic mice exhibited overgrowth of lymphatic vessels but not blood vessels, underscoring its specific role in lymphangiogenesis.^373^ Subsequently, VEGF-D was identified and shown to also promote lymphangiogenesis.^73,374^ However, while *Vegfc*-deficient mouse embryos failed to form lymphatic vessels, this defect could be rescued by external VEGF-C or -D supplementation.^375^ Interestingly, although VEGF-D is a potent lymphangiogenic factor, it is not essential for lymphatic development.^376^ VEGFR3 is crucial for blood and lymphatic vascular formation during early developmental stages. Studies involving mutations in the ligand-binding domain or tyrosine KD of VEGFR3 revealed that ligand binding is essential for lymphatic vessel development but not for blood vessel development. This supports a regulatory model where VEGFR3 modulates VEGF-A/VEGFR2 signaling through VEGFR2/3 heterodimers for blood vascular formation. Thus, the VEGF-C/D-VEGFR3 axis plays a pivotal role in lymphangiogenesis, distinct from its tole in blood vessel development.^377^ Furthermore, other members of the VEGF/VEGFR family also contribute significantly to lmphangiogenesis. LEC-specific knockout of *Vegfr2* (*Flk1*) leads to hypoplastic yet functional lymphatic vessels, indicating that VEGFR2, activated by both VEGF-A and VEGF-C, plays a role in this process. Additionally, VEGF-A alone has been shown to induce lymphangiogenesis,^378,379^ although this effect is partially mediated by macrophage recruitment.^380,381^ Thus, while VEGF-C/D-VEGFR3 remains central to lymphangiogenesis, VEGFR2 and VEGF-A also play supportive roles, often involving indirect mechanisms such as macrophage involvement.

Ang-TIE signaling,^346,382^ FGF signaling,^342,383^ and other factors including HGF,^384^ PDGF,^385^ and IGF^386^ have been shown to promote lymphangiogenesis.^372,387^ However, despite the identification of numerous lymphangiogenic factors, it remains unclear whether the mechanisms of lymphatic vessel sprouting and guidance resemble those of angiogenesis. The current model divides lymphangiogenesis into four key steps: (1) guidance/alignment, (2) proliferation, (3) sprouting, and (4) redirection.^372^ In this process, the BM stabilizes lymphatic vessels,^388^ LEC tip cells guiding sprouting,^389^ and tissue macrophages provide VEGF-C guidance.^390^ Future studies should focus on dissecting each step of lymphangiogenesis and uncovering the molecular mechanisms underlying these processes.

#### Embryonic development

Blood vessels are present in nearly all tissues, making VEGF signaling crucial for regulating angiogenesis and maintaining tissue homeostasis, including vascular permeability. To highlight its physiological significance, VEGF signaling plays key roles in (1) embryonic development by regulating angiogenesis, (2) maintaining endocrine organ homeostasis through vascular permeability, and (3) exerting neurotrophic functions independent of ECs.

Mouse embryos lacking VEGF ligands or their receptors exhibit embryonic lethality due to vascular defects (Table 1).^358–362,375^ Specifically, VEGF-A, VEGFR2, and VEGFR3 promote hematopoietic and EC development, while VEGFR1 ensures precise blood vessel assembly. In contrast, PlGF and VEGF-B have minimal roles in development^87,391^ whereas VEGF-C drives lymphatic vascular formation. Even slight alterations in VEGF levels, such as deletion of a single allele or modest overexpression, lead to abnormal blood vessel development and embryonic lethality,^358,359^ emphasizing the need for precise VEGF signaling regulation during embryonic development. Postnatally, VEGF signaling is critical for organ development, as its disruption causes glomerular defects in neonates,^392^ but not in adult mice.^393^ Although VEGF/VEGFR signaling is vital for embryonic development, it remains active in adults, affecting both pathological and physiological conditions. For instance, modestly elevated circulating VEGF levels from early adulthood have been shown to preserve a youthful microvascular phenotype and extend lifespan in mice.^394^ Thus, while VEGF signaling is indispensable during development, its regulation continues to play a beneficial role in maintaining physiological health throughout life.

### Vascular permeability

Vascular permeability is a selective mechanism that regulates material exchange between blood vessels and surrounding tissues through small and large pores,^395^ glycocalyx, and tight junctions on ECs (Fig. 8b).^396^ Under normal conditions, the endothelium allows the passage of solutes and small molecules while restricting larger molecules. However, in response to stimuli, the endothelium can become more permeable, enabling the leakage of larger molecules or cells. This permeability varies across organs based on physiological needs; for example, fenestrated endothelial in endocrine glands and kidney peritubular capillaries facilitate rapid transport, while the blood–brain barrier features tight junctions and perivascular coverage to restrict permeability. This plasticity is tightly controlled by various extracellular signaling molecules, including VEGF, Ang, histamine, platelet-activating factor, as well as intracellular mediators, such as NO, focal adhesion kinase (FAK), and GTPases.^397,398^

VEGF-A/VEGFR2 signaling is widely recognized for promoting vascular permeability through multiple mechanisms. Specifically, the Y949 (Y951 in humans) in VEGFR2 in mice binds to TSad, regulating c-SRC signaling at the EC junction.^399^ Disruption of Y949 signaling stabilizes adherens junctions, blocking extravasation.^171^ In contrast, the Y1173 in mice (Y1175 in humans) binds to PLCγ1, driving EC proliferation,^400^ and its disruption halts developmental processes.^401^ Notably, the role of VEGFR2 in permeability depends on its co-receptor NRP1, as SRC signaling in EC is largely NRP1-dependent.^189^ Beyond direct junction regulation, VEGFR2 activates AKT and eNOS to promote NO synthesis,^402^ indirectly enhancing vascular permeability, a process regulated by caveolin-1.^403,404^ These findings highlight the critical role of VEGF-A/VEGFR2 in paracellular permeability, surpassing even certain inflammatory cytokines in potency.^171^ Additionally, VEGF stimulates the formation of vesiculo-vacuolar organelles (VVO) in EC,^405^ interconnected structures spanning the EC lumen, which support transcellular permeability and macromolecular extravasation. Thus, VEGF signaling plays a dual role in regulating both paracellular and transcellular permeability, underscoring its importance in vascular function.^406^ VEGF/VEGFR signaling primarily regulates endothelial permeability through VEGFR2, facilitating the rapid extravasation of macromolecules (Fig. 8b). Furthermore, the glycocalyx, a key structural component for vascular permeability, can be cleaved by inflammatory cytokines.^407^ While it remains unclear whether VEGF signaling directly regulates the glycocalyx in EC, the glycocalyx itself inhibits VEGFR2 internalization, reducing VEGF-induced vessel permeability.^408^ Overall, these interactions highlight the complex relationship between VEGF signaling and the glycocalyx in regulating vascular function.

Under physiological conditions, most tissues require a certain degree of vascular permeability for efficient substance exchange. Particularly, endocrine organs exhibit high permeability to facilitate hormone delivery, maintained by both general and organ-specific factors.^409^ For example, the thyroid gland, the largest endocrine organ,^410^ features a dense capillary network with abundant endothelial fenestrae,^410^ which are VEGF-dependent for their maintenance.^411^ Recent studies highlight that VEGF/VEGFR signaling, rather than Ang/TIE signaling molecules, is prominently expressed in thyroid organs.^412^ Blocking VEGF or VEGFR2 in the thyroid leads to thickened cytoplasmic membrane, loss of EC fenestration, and accumulation of caveolae, modestly reducing circulating hormone levels.^412,413^ Importantly, VEGF-VEGFR2 inhibition not only alters vascular permeability but also causes significant vascular regression in thyroid, more pronounced than tumors.^413,414^ Clinically, this is evident as bevacizumab rapidly reduces thyroid perfusion.^415^ Even in healthy adults, autocrine VEGF is essential for blood vessel homeostasis, as endothelial-specific deletion of *Vegf* results in progressive endothelial degeneration.^416^ Thus, endocrine organs uniquely depend on VEGF-VEGFR2 signaling not only for vascular permeability but also for maintaining vascular homeostasis.

### Animal models of VEGF and VEGFR

Animal models, including various VEGF and VEGFR knockout and conditional knockout mouse lines, have provided pivotal insights into the complexities of VEGF signaling in endothelial and lymphatic cells. These models reveal how genetic alterations in the VEGF/VEGFR pathways affect vascular development, lymphangiogenesis, and pathological processes, offering a detailed view of the VEGF regulatory network in both physiological and pathological contexts. These distinct phenotypes underscore the crucial roles of VEGF signaling in various biological processes (Table 1).

## VEGF and VEGFR signaling in diseases

The discovery of VEGF has been a pivotal milestone and has significantly advanced our understanding of angiogenesis. Recent studies have investigated the specific roles of different VEGF isoforms under various conditions. These isoforms have also been found to contribute to several cardiovascular, ocular, metabolic, and reproductive disease. Despite this advancement in our knowledge, the intricate relationship between hyperglycemia, oxidative stress, inflammation, and VEGF expression remains a challenging area of research.

### Cancer

Cancer, the second leading cause of death worldwide, is projected to account for approximately 618,120 fatalities and 2 million new cases in the US in 2025, according to the American Cancer Society’s latest estimates.^417^ Globally, the burden continues to escalate, with projections suggesting new cancer cases could surpass 35 million by 2050. This complex disease can originate in nearly any part of the body, characterized by the uncontrolled proliferation of abnormal cells that invade neighboring tissues and metastasize to distant sites through blood vessels, reflecting its developmental intricacy and diverse manifestations.

#### Tumor angiogenesis

**Fig. 9:**
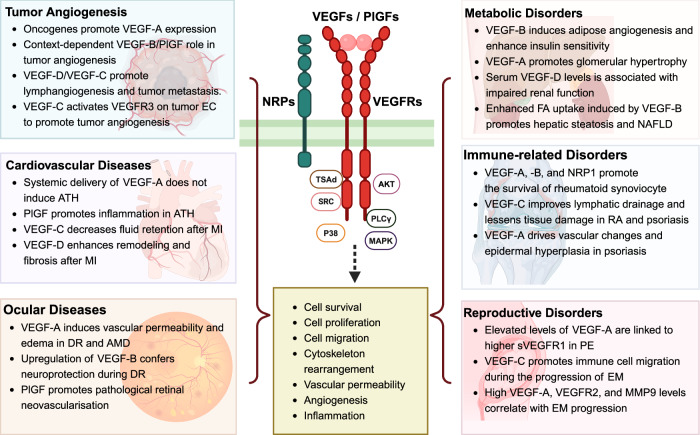
VEGF/VEGFR signaling under pathological conditions. The diverse roles of VEGF ligands and their receptors drive pathological processes, including tumor angiogenesis, cardiovascular diseases, ocular diseases, and metabolic/immune-related/reproductive disorders. Key molecular interactions and downstream signaling pathways are upregulated or dysregulated in disease states, thereby promoting cell survival, migration, and proliferation. These pathways highlight the therapeutic potential of targeting VEGF/VEGFR signaling across various disease contexts. ATH atherosclerosis, MI myocardial ischemia, DR diabetic retinopathy, AMD age-related macular degeneration, FA fatty acid, NAFLD non-alcoholic fatty liver disease, RA rheumatoid arthritis, PE pre-eclampsia, EM endometriosis. Created in BioRendender.com

Dr. Folkman proposed the existence of a tumor angiogenic factor (TAF) critical for tumor growth, suggesting that inhibiting TAF could suppress tumor progression.^7^ Angiogenesis, essential for solid tumor growth and metastasis, is regulated by a balance of pro- and anti-angiogenic factors.^418^ Tumor cells promote angiogenesis by secreting VEGFs, stabilized by HIF-1α in a hypoxic TME,^84,419^ while oncogenes such as RAS and RAF (Fig. 9) further affect VEGF expression.^420–422^ In turn, VEGF from tumor and stromal cells drives pathological angiogenesis by activating VEGFR1 and VEGFR2 on tumor ECs.^423^

Recent studies show that most VEGF family members, except PlGF, correlate with angiogenesis and lymphangiogenesis in cancer.^424^ However, the role of PlGF remains complex, as it can either promote^425,426^ or inhibit tumor growth^427,428^ depending on the context. Similarly, VEGF-B exhibits varying effects on tumor angiogenesis and survival, with its dual functions linked to FGF-2/FGFR activity.^137^ Likewise, VEGF-C, activating VEGFR2 and VEGFR3, promotes blood vessel growth in breast cancer, while its overexpression enhances lymphangiogenesis and lymphatic metastasis.^61,429–431^ In addition, VEGF-A also supports lymphangiogenesis by activating VEGFR2, forming heterodimers with VEGFR3, and recruiting macrophages and mast cells to secrete VEGF-C and VEGF-D, thus promoting lymphatic vessel growth.^33,432^ Furthermore, inhibition of VEGFR3 signaling blocks VEGF-C-induced lymphangiogenesis and tumor cell infiltration into lymphatic vessels.^433^ Notably, VEGF-D overexpression can drive tumor growth and angiogenesis, enabling to anti-VEGF-A therapy and highlighting its critical role in cancer progression.^434,435^ These findings underscore the complex and pivotal roles of VEGF family members in tumor angiogenesis, lymphangiogeneis, and therapeutic evasion.

Importantly, a recent study presents a single-cell atlas of tumor vasculature, analyzing around 200,000 cells from various cancers.^349^ Specifically, tumor angiogenesis is traced from venous ECs to capillary and arterial ECs through angiogenic stages, with apelin+ tip cells identified as biomarkers for poor prognosis and anti-VEGF therapy response. Furthermore, LECs show two lineages, one driving lymphangiogenesis and another enhancing antigen presentation. Additionally, BASP1+ matrix-producing pericytes linked to endoplasmic reticulum stress were shown to play a pro-angiogenic role. Moreover, neovascular ECs also contribute to an immunosuppressive TME through stromal and immune cell interactions. In summary, these findings highlight the interplay between vascular cells and the TME, offering insights into vascular heterogeneity and novel anti-angiogenic therapeutic targets.

### Cardiovascular diseases

Cardiovascular diseases (CVDs) encompass a range of disorders affecting the heart and blood vessels, rooted in vascular dysfunction, including atherosclerosis, myocardial infarction, and hypertension.

#### Atherosclerosis

VEGF plays a complex role in atherosclerosis, with both beneficial and detrimental effects. Systemic VEGF gene delivery does not induce atherosclerosis in hypercholesterolemic mice,^436^ but local administration of VEGF-A and VEGF-D promotes angiogenesis and intimal hyperplasia in rabbits.^437^ Moreover, VEGF can recruit macrophages and monocytes, potentially exacerbating plaque formation,^438^ as demonstrated by intracoronary VEGF-A164 delivery, which worsened arteriosclerotic injury in rabbits. Similarly, macrophages in human plaques express VEGF-A via HIF-1α,^439^ highlighting its inflammatory role. Additionally, PlGF-2 gene transfer increases VEGF-A levels, driving inflammatory angiogenesis,^440^ while PlGF itself promotes atherosclerosis in hypercholesterolemic models despite reducing macrophage content.^441,442^ Furthermore, VEGF facilitates macrophage infiltration through VEGFR1 and mobilizes endothelial and smooth muscle progenitor cells via VEGFR2, contributing to coronary arteriosclerosis.^443,444^ Additionally, VEGF-A accelerates vascular remodeling by enhancing T cell recruitment to vessels.^445^ Therefore, while VEGF supports angiogenesis and vascular repair, its role in plaque formation and progression underscores its context-dependent and multifaceted impact on atherosclerosis (Fig. 9).

Low VEGF-C levels are linked to higher mortality in patients with coronary artery disease and may enhance plaque stability, positioning VEGF-C as a promising biomarker and therapeutic target for CVD. Specifically, its protective effects on smooth muscle cells, promotion of cholesterol efflux, and reduction of cellular stress underscore its role in maintaining vascular health and mitigating the progression of atherosclerosis.^446,447^ Thus, further investigation is warranted to explore its clinical applications for risk stratification and innovative treatments. Similarly, VEGF-D is expressed at all stages of human atherosclerotic lesion development, implicating its involvement in the disease.^448^ For example, adenovirus-mediated VEGF-D gene transfer has been shown to reduce neointimal thickening and macrophage infiltration, suggesting its therapeutic potential.^449^ Moreover, elevated VEGF-D levels also independently predict mortality in patients with coronary artery disease undergoing elective angiography.^450^ Collectively, these findings emphasize multifaceted role of VEGF-D in atherosclerosis, highlighting its potential as both a therapeutic target and a prognostic marker in CVD. Together, VEGF-C and VEGF-D represent critical factors in understanding and managing cardiovascular diseases.

#### Myocardial ischemia (MI)

The roles of VEGFs and VEGFRs in MI are complex, significantly impacting cardiac function and recovery. Specifically, ischemia triggers VEGF release from cardiomyocytes, binding to VEGFR1 and VEGFR2 on microvascular ECs,^17,451,452^ promoting angiogenesis and vascular remodeling. However, recent studies show mild hypoxia in developing mouse hearts does not induce VEGF-A or coronary angiogenesis,^453^ suggesting other factors. Moreover, *Vegfa* deletion in cardiomyocytes impairs blood vessel formation and worsens heart contractions.^454^

Inflammation drives MI, with Annexin A1 promoting cardiac repair and angiogenesis via macrophage-derived VEGF-A.^455^ Similarly, VEGFR2 signaling also aids barrier function with losartan treatment reduces ischemic reperfusion injury in mice.^456^ Intracoronary VEGF-A gene transfer improves myocardial perfusion, offering a strategy for therapeutic angiogenesis in ischemic heart disease.^457^ Furthermore, VEGF-B or PlGF overexpression enhances coronary vasculature and stimulates cardiomyocyte hypertrophy, yet long-term hypertrophy effects remain unclear.^56,458,459^ Combining PlGF and VEGF-A enhances angiogenesis in ischemic myocardial tissue resistant to VEGF-A alone, suggesting synergistic benefits in certain clinical scenarios.^162^ Notably, VEGF-B promotes coronary blood vessel development without permeability or inflammation, making it an attractive therapeutic target.^56,460^

Post-MI, VEGF-C enhances lymphangiogenesis, reduces fluid retention and inflammation (Fig. 9), and improves cardiac function.^461,462^ Additionally, VEGF-C protects cardiomyocytes by activating AKT signaling through VEGFR2, thereby reducing apoptosis.^463^ Similarly, VEGF-D increases vascular permeability, stimulates angiogenesis, and improves blood flow, yet it also contributes cardiac fibrosis, underscoring the need to balance its therapeutic benefits and adverse effects.^464,465^ In summary, the VEGFs and their receptors diversely affect MI through angiogenesis, inflammation, cardiac repair, necessitating deeper understanding for targeted therapies.

#### Hypertension

Hypertension is the most common comorbidity associated with anti-VEGF therapy.^466,467^ Under hypertensive conditions, disrupted VEGF signaling impairs endothelial dysfunction and alters hemodynamics. Specifically, VEGF-A binding to VEGFR2 or shear stress activates the PI3K/AKT pathway, stimulating eNOS to produce vasodilators such as NO and prostacyclin (PGI2), highlighting the critical role of VEGF signaling in vascular tone regulation.^468,469^

Studies in animal models and patients treated with VEGF signaling pathway (VSP) inhibitors show reduced urinary nitrite/nitrate excretion and serum NO metabolites, impairing NO-dependent microvasodilation.^470,471^ Additionally, VSP inhibitors upregulate endothelin-1 (ET-1), a potent vasoconstrictor, contributing to hypertensive effects. These findings reveal the complex interplay between VEGF signaling, NO production, and blood pressure regulation.^472,473^ In patients with gastrointestinal stromal tumors, regorafenib-induced ET-1 fluctuations correlated with therapy cycles and blood pressure changes.^474^ In contrast, sorafenib did not significantly alter ET-1 levels in renal cell carcinoma patients, despite hypertension.^475^ Similarly, preclinical studies in rats showed that sunitinib increased ET-1 and blood pressure, effects reversed by the endothelin receptor antagonist macitentan.^472^ These results suggest a variable interplay between VSP inhibitors, ET-1, and blood pressure, depending on the inhibitor and cancer type. Moreover, VSP inhibition may trigger hypertension via microvascular rarefaction, reducing vessel density, or functional rarefaction, heightening vasomotor tone and limiting blood flow.^476^ Recent studies also link VSP inhibitors to salt-sensitive hypertension through interstitial sodium buildup and osmotic stress, especially in the skin.^477–479^ This complex interplay between VEGF signaling, endothelial function, and blood pressure highlights challenges in managing anti-VEGF therapy-induced hypertension, warranting further research for effective strategies.

### Ocular diseases

Ocular diseases encompass a range of conditions that target the eye and its visual system, significantly impacting various retinal cell types and structures. Notably, the prevalence of ocular microvascular diseases, such as DR and AMD has risen considerably. These conditions pose significant threats to ocular health and visual acuity, often leading to visual impairment or blindness if not treated promptly.

#### Diabetic retinopathy

The global rise in diabetes has intensified microvascular complications, notably, DR, a leading cause of vision.^480,481^ Specifically, hyperglycemia forms AGEs, driving neovascularization, permeability, and macular edema in DR.^482^ As a result, retinal cells such as ganglion cells,^483^ astrocytes,^484^ ECs,^484^ pericytes,^485^ microglia,^486^ Müller glia,^487^ and retinal pigment epithelial (RPE) cells^488^ secrete VEGFs. Moreover, ischemia worsens inflammation and endothelial damage, upregulating VEGFs, VEGFRs, and HIF-1α to regulate neovascularization.^489–491^

VEGF-A plays a dual role in DR, both promoting vascular permeability and protecting retinal cells.^492^ Initially, it increases edema, and disrupts blood-retinal barrier,^493,494^ fueling endothelial proliferation and proliferative diabetic retinopathy (PDR), marked by abnormal retinal vessel growth.^495^ Yet, it also guards neurons and Müller cells against apoptosis,^483,496^ reflecting complex regulation.^497^

Hyperglycemia-induced reactive oxygen species (ROS) abnormally phosphorylate VEGFR2 independently of VEGF-A,^498^ with elevated VEGFR2 expression in diabetic retinal microvascular ECs, especially in the macula.^499^ Consequently, VEGF-A phosphorylates tight junction proteins such as occludin, increasing permeability,^500–502^ and activates pathways such as phospholipase A2,^503^ PKC,^504^ and PI3K/AKT,^505^ driving endothelial dysfunction and plasma extravasation. Additionally, VEGF-A level variations affect DR risk,^506,507^ with elevated VEGF-A, PlGF, and VEGF-B in vitreous and serum promoting angiogenesis and PDR.^508,509^ However, their roles in progression and treatment response remain unclear.

In contrast, VEGF-B, VEGFR1, and NRP1 provide neuroprotection via AKT and ERK pathways, reducing apoptosis,^510^ while low VEGF-B impairs vascular integrity by downregulating VE-cad, ZO-1, and CDC42.^511^ Similarly, PlGF rises in diabetic macular edema (DME) and PDR,^512–514^ disrupting RPE barrier integrity via MEK/ERK under hypoxia.^515^ For instance, PlGF deletion in diabetic mice reduces apoptosis and microcapillary damage by inhibiting HIF-1α-induced VEGF signaling through AKT,^516^ yet its overexpression increases microaneurysms, leakage, and inflammation via IL-6.^517^ Additionally, dysfunctional lipid metabolism upregulates VEGF-A, VEGF-C, VEGF-D, and PlGF, advancing DR.^518^ Notably, VEGFR2 localizes to leaky microvessels, while VEGFR3 is limited to specific PAL-E-positive vessels, indicating altered receptor expression.^519^ Although VEGFs’ roles in DR are clear, isoform-specific contributions and receptor crosstalk require further study to refine targeted therapies.

#### Age-related macular degeneration

AMD is a complex, multifactorial disease influenced by aging, environmental factors, and genetic predisposition. Its pathogenesis is associated with chronic inflammation, lipid accumulation, oxidative stress, and ECM dysfunction.^520^ In neovascular (wet) AMD (nAMD), immune cell recruitment to the macula leads to the release of pro-inflammatory and pro-angiogenic cytokines, including VEGF, driving pathological neovascularization.

VEGF-A is the primary factor mediating this process, making it a key therapeutic target.^521^ Additionally, VEGF-B overexpression disrupts the outer blood-retinal barrier and promotes inflammatory angiogenesis by upregulating cell survival factors.^522^ Notably, patients with wet AMD and polypoidal choroidal vasculopathy exhibit elevated VEGF-B levels, suggesting its involvement in disease progression, particularly after VEGF-A inhibition.^523^

Beyond VEGF-A and VEGF-B, VEGF-C and VEGF-D, traditionally recognized for their roles in lymphangiogenesis via VEGFR-3, have recently been implicated in retinal and choroidal angiogenesis.^524,525^ These factors can activate VEGFR-2, contributing to vascular permeability and neovascularization independently of VEGF-A. Importantly, VEGF-C and VEGF-D are upregulated following VEGF-A inhibition with agents such as aflibercept or bevacizumab, which may limit the efficacy of anti-VEGF-A therapy and lead to persistent disease activity.^526^ RPE cells from wet AMD patients express VEGF-C and VEGF-D, further promoting neovascularization despite the absence of lymphatic vessels.^524^ Inflammatory cytokines in AMD also upregulate VEGF-A and VEGF-C, with VEGFR-2 and VEGFR-3 detected in choroidal neovascularization (CNV) membranes, reinforcing the role of VEGF family members in disease progression.^527,528^ Given the limitations of VEGF-A inhibition alone, novel approaches targeting VEGF-C and VEGF-D have emerged. A phase 2b clinical trial of OPT-302, a VEGF-C/D inhibitor, demonstrated that adding VEGF-C/D blockade to standard anti-VEGF-A therapy (ranibizumab) significantly improved visual outcomes in nAMD patients.^529^ These findings suggest that dual VEGF-A and VEGF-C/D inhibition may enhance therapeutic efficacy by targeting alternative angiogenic pathways, although further studies are needed to confirm long-term benefits.

Meanwhile, VEGFR1, highly expressed by infiltrating monocytes and macrophages in AMD, modulates angiogenesis by suppressing VEGF-A-induced migration when neutralized.^530^ Blocking PlGF and VEGFR1 reduces monocyte recruitment after laser-induced injury, highlighting VEGFR1’s role in immune cell infiltration.^531,532^ Additionally, excessive VEGF-A disrupts RPE barrier function, exacerbating choroidal neovascularization.^533^ However, while VEGF-A/VEGFR-2 inhibition reduces vascular permeability, RPE-derived VEGF-A is essential for choriocapillaris maintenance,^534,535^ underscoring the need for a delicate balance in anti-VEGF therapies to suppress pathological signaling without compromising retinal function.

A comprehensive understanding of VEGF signaling in AMD, particularly the interplay between VEGF-A, VEGF-B, VEGF-C, and VEGF-D, is essential for optimizing therapeutic strategies and refining treatment approaches to improve long-term patient outcomes.

### Metabolic diseases

Metabolic disorders arise from disruptions in energy utilization and storage, driven by hormonal, enzymatic, and cellular imbalances. In this context, VEGFs play a dual role in metabolic diseases, influencing vascular complications in diabetes mellitus, diabetic nephropathy, and non-alcoholic fatty liver disease with both protective and detrimental effects.

#### Type 2 diabetes mellitus (T2DM)

T2DM is a metabolic disorder driven by insulin resistance or β-cell dysfunction, leading to vascular alterations in insulin-sensitive tissues.^536^ Notably, VEGF-A, primarily produced by pancreatic α and β cells, plays a key role in islet microvasculature.^537^ Deleting *Vegf* in insulin-producing cells reduces islet vascularization and impairs insulin secretion, while VEGF-A deficiency leads to islet hypoxia and elevated blood glucose levels.^537–539^

Glucose homeostasis and VEGF-A secretion are tightly linked. Lower glucose levels reduce VEGF-A secretion, whereas prolonged hypoglycemia induces apoptosis in ECs and β cells.^540^ Exogenous VEGF-A mitigates hypoglycemia-induced damage, suggesting potential therapeutic applications.^541^ However, chronic VEGF upregulation may be detrimental, contributing to β-cell dysfunction and PKC-mediated endothelial abnormalities in diabetic vasculopathy.^542,543^ In the inflammatory context, VEGF-A exerts complex effects. Diabetic patients exhibit impaired monocyte responses to VEGF-A, hindering inflammatory cell infiltration in ischemic tissues and compromising wound healing.^544^ Conversely, VEGF-A promotes macrophage recruitment, supporting β-cell proliferation and regeneration.^545–547^ Blocking VEGF-B improves glucose tolerance (Fig. 9) and insulin sensitivity,^548^ while VEGF-C accelerates wound healing in diabetic conditions.^549^

Ultimately, VEGF signaling acts as a double-edged sword in T2DM. While essential for islet function, dysregulated VEGF expression may worsen β-cell dysfunction and vascular complications. Future research should clarify the roles of VEGF isoforms to develop targeted therapies that optimize VEGF levels, improving glycemic control and preventing diabetic complications.

#### Diabetic nephropathy (DN)

DN, a major renal complication of diabetes, arises from chronic hyperglycemia-induced oxidative stress, inflammation, and vascular dysfunction.^550,551^ In the kidney, VEGF-A, mainly produced by podocytes and detected in distal tubules and collecting ducts,^552–554^ is expressed in glomerular and tubular cells such as pericapillary ECs, mesangial cells, and interstitial fibroblasts, to maintain vascular integrity.^519,555,556^ Initially, in early DN, rising VEGF-A levels in renal and urinary systems increase permeability and endothelial dysfunction, driving glomerular damage,^557,558^ with elevated VEGF and VEGFR2 expression in diabetic models.^559,560^ For example, in eNOS (*Nos3*) knockout mice, higher VEGF-A correlates with glomerular hypervascularization, worsening DN.^561^ However, anti-VEGF therapy reduces albuminuria and glomerular dysfunction in diabetic mice,^562,563^ although excessive suppression risks endothelial injury and increased permeability.^564^

VEGF-B contributes to DN by inducing podocyte insulin resistance via lipid accumulation,^565^ with elevated levels in T2DM patients linked to renal decline.^566^ Notably, an anti-VEGF-B/IL-22 fusion protein mitigates oxidative stress, inflammation, and lipid deposition, offering therapeutic potential.^567^ Similarly, VEGF-C and VEGF-D, signaling through VEGFR3, regulate glomerular permeability despite the absence of lymphatics in glomeruli,^568^ as podocyte-derived VEGF-C enhances permeability via VEGFR3 and VEGFR2.^569,570^ In contrast, podocyte-specific VEGF-C overexpression in diabetic mice improves albuminuria and endothelial function by lowering VEGFR2,^571,572^ while elevated VEGF-D correlates with renal impairment, albuminuria, and proteinuria.^573^

Nevertheless, excessive VEGF manipulation can be detrimental. Complete *Vegfa* loss in podocytes causes neonatal death, and a single allele leads to proteinuria and endotheliosis,^392^ while its deficiency disrupts complement regulation, increasing C3 accumulation.^574^ Likewise, bevacizumab-induced VEGF inhibition in humans causes endothelial injury and plasma buildup,^575^ and *Vegfr1* deletion in podocytes triggers severe proteinuria, although kinase-deficient VEGFR1 mitigates this, suggesting sVEGFR1’s protective role.^166^

Thus, VEGF signaling in DN balances vascular protection and pathological remodeling. The complex interplay of VEGF isoforms and receptors highlights the need for precise therapies to modulate VEGF activity, preserve renal function, and minimize harm, necessitating further research.^576–578^

#### Non-alcoholic fatty liver disease (NAFLD)

NAFLD is a major public health concern characterized by excessive hepatic lipid accumulation in individuals with minimal or no alcohol consumption.^579^ It encompasses a spectrum from simple steatosis to non-alcoholic steatohepatitis (NASH), which can progress to fibrosis, cirrhosis, and HCC.^580^ The advancement of liver fibrosis is marked by increased vascular growth, as observed in both human and animal models.^581^ Recent findings suggest a potential role for VEGF in NAFLD pathogenesis and progression.^582,583^ Notably, VEGF and VEGFR1 mRNA levels are elevated in steatotic livers compared with NASH, indicating an early induction of angiogenesis in NAFLD.^584^ However, clinical studies have shown conflicting results regarding VEGF-A and VEGFR alterations in NAFLD progression.^584,585^

Hepatocytes play a key role in NAFLD progression by synthesizing VEGF-A, which promotes fibrosis and endothelial dysfunction, contributing to disease severity.^586^ In a murine model of diet-induced NASH, VEGFR2 inhibition reduced steatosis and inflammation, highlighting VEGF signaling as a potential therapeutic target.^587^ Beyond hepatic angiogenesis, adipose tissue metabolism affects NAFLD development. VEGF-B signaling enhances fatty acid uptake in the liver, driving hepatic steatosis in diabetic models.^588^ Additionally, VEGF-B has been identified in the subcutaneous white adipose tissue of NAFLD patients, emphasizing its translational relevance in human disease.^589^ Patients with NAFLD also exhibit elevated serum levels of angiogenic markers, including VEGF, sVEGFR1, and sVEGFR2, which correlate with hepatic fibrosis severity and may serve as biomarkers.^584^

Emerging evidence suggests VEGF-C as a therapeutic target for NAFLD, with flavonoid-based interventions showing potential in modulating VEGF-C activity.^590^ The interplay between VEGF-A, VEGF-B, and VEGF-C contributes to hepatic lipid accumulation, fibrosis, and carcinogenesis, highlighting VEGF signaling as a critical target for therapeutic strategies in NAFLD.

### Immune-related diseases

Immune-related diseases are characterized by dysregulation of the immune system, resulting in inflammation and tissue damage. VEGFs are critical in these conditions by promoting angiogenesis and modulating immune responses.

#### Rheumatoid arthritis (RA)

RA is a chronic autoimmune disorder characterized by persistent synovial inflammation, leading to joint damage and functional impairment.^591^ A key driver of RA progression is angiogenesis, which facilitates inflammatory cell infiltration and pannus formation, accelerating joint destruction.^592^ Notably, HIF-1 and HIF-2 are highly expressed in the RA synovium, promoting VEGF production and excessive vascularization, which exacerbate inflammation and synovial hyperplasia.^593^

VEGF-A plays dual roles as both a pro-inflammatory mediator and an angiogenesis promoter within synovial tissue.^594^ Synovial macrophages and fibroblasts produce VEGF, while osteoclasts and their precursors express VEGF receptors, forming a complex network that drives disease progression.^595^ Elevated serum VEGF levels correlate with c-reactive protein (CRP), highlighting its diagnostic and prognostic value in RA.^596,597^ Furthermore, VEGF supports rheumatoid synoviocyte survival and proliferation through IL-6/JAK2/STAT3 and Notch signaling, reinforcing its role in RA pathogenesis.^598^

Other VEGF family members contribute to RA pathology.^599^ VEGF-B deficiency reduces synovial angiogenesis and inflammation, whereas VEGF-C promotes lymphangiogenesis, improving lymphatic drainage and reducing tissue damage.^600^ Given the central role of VEGF signaling, targeted therapies such as soluble VEGFRs, anti-VEGF antibodies, and the VEGFR2 inhibitor ramucirumab have shown promise in reducing inflammation and disease severity.^601–603^ Their efficacy is further enhanced in combination with methotrexate, presenting a potential therapeutic strategy for managing RA.

#### Psoriasis

Psoriasis is a chronic inflammatory skin disorder that significantly impacts the quality of life.^604^ A key but underappreciated factor in its pathogenesis is aberrant angiogenesis, which increases vascular permeability, capillary dilation, and elongation, contributing to epidermal hyperplasia and disease progression.^605,606^ Elevated VEGF-A levels in psoriatic epidermis and plasma promote EC proliferation, migration, and survival while enhancing vasodilation and permeability.^607^ VEGF-A also regulates keratinocyte proliferation and differentiation, with VEGFR1, VEGFR2, and NRPs expressed in keratinocytes, indicating autocrine VEGF-A signaling.^608,609^

Non-lesional psoriatic skin overexpresses VEGF isoforms such as VEGF-A, VEGFR3, and sNRP1.^610,611^ VEGFR1, VEGFR2, and VEGFR3 are upregulated in psoriatic lesions, while VEGF-C, in conjunction with nerve growth factor (NGF), has been implicated in disease development.^612^ Preclinical models highlight the role of VEGF in psoriasis pathogenesis (Fig. 9). Transgenic VEGF delivery induces chronic inflammation with hyperplasia and vascular abnormalities resembling psoriasis.^613^ Excess VEGF-A increases vascular density and permeability, exacerbating inflammation. In psoriasis mouse models, anti-VEGF therapies reduce inflammation, normalize epidermal structure, and decrease vascular density and immune infiltration.^614^

Targeting angiogenesis through anti-VEGF therapies holds potential for psoriasis treatment, yet further clinical studies are required to assess their efficacy and safety. A better understanding of the angiogenesis-inflammation interplay may lead to improved therapeutic strategies and better patient outcomes.

### Reproductive disorders

Reproductive disorders, a broad spectrum of conditions that affect male and female reproductive health, significantly affect hormone production, gamete formation, and reproductive capacity. VEGFs play a crucial role in the development and maintenance of reproductive tissues, including follicular development, corpus luteum formation, and endometrial vascularization.

#### Pre-eclampsia (PE)

PE is a significant hypertensive disorder arising after the 20th week of gestation, often accompanied by complications such as proteinuria, maternal organ dysfunction, or uteroplacental dysfunction.^615^ A key pathological feature is the disruption of angiogenic balance due to trophoblast dysfunction and incomplete spiral artery remodeling, leading to placental hypoxia, oxidative stress, and endothelial dysfunction.^616^

Endovascular trophoblasts and decidual leukocytes regulate VEGF and PlGF production in normal pregnancies.^617^ Free VEGF helps counteract endothelial shear stress and inflammation, maintaining vascular quiescence.^618^ However, PE is marked by elevated plasma sVEGFR1 levels before clinical diagnosis.^619^ Placental hypoxia triggers VEGF secretion,^205^ which in turn increases sVEGFR1 expression, disrupting angiogenic balance.^620^ Experimental models confirm that sVEGFR1 infusion induces PE-like features, including hypertension and proteinuria.^621^ Similarly, adenoviral sVEGFR1 overexpression reduces VEGF and PlGF, impairing placental and fetal growth.^622^ Clinically, placental sVEGFR1 levels are markedly elevated in PE, antagonizing VEGF and PlGF, which leads to systemic endothelial dysfunction.^622^ The severity of PE correlates with sVEGFR1 elevation, emphasizing the critical VEGF-sVEGFR1 balance.^623^ The sVEGFR1:PlGF ratio serves as a predictive marker for PE risk.^105,624^ Additionally, the sVEGFR1-e15a variant predominates in PE circulation, highlighting its role in disease pathogenesis.^625^

Targeting VEGF and PlGF have been explored in experimental PE models. In sVEGFR1-induced PE, recombinant VEGF-A121 administration lowered blood pressure without affecting proteinuria.^626^ Similarly, in the reduced uterine perfusion pressure model (widely used animal model of pre-eclampsia), rhVEGF-A121 and recombinant PlGF reduced sVEGFR1 levels, improved renal function, and mitigated oxidative stress.^627–629^ Furthermore, adenoviral VEGF expression in hypertensive mouse models decreased blood pressure and proteinuria.^630,631^ Notably, modified VEGF-B conjugated with an elastin-like polypeptide showed therapeutic potential without inducing angiogenesis.^632^

Despite promising results, VEGF-based therapies raise safety concerns. Excess VEGF can cause in utero mortality due to cardiac failure, and its placental transfer remains unclear.^633^ The potential fetal risks highlight the need for rigorous safety evaluations before clinical application. Careful translation of VEGF-targeted therapies from preclinical models to clinical practice is essential for effective PE management.

#### Endometriosis

Endometriosis is a persistent gynecological disorder characterized by the ectopic growth of endometrial-like tissue, primarily in the pelvic region.^634^ Its pathogenesis involves endocrine, inflammatory, and pro-angiogenic factors, although their precise role—whether causal or secondary—remains debated.^635^ Notably, angiogenesis is a key driver of lesion progression.^636^

VEGF is regulated by multiple signaling pathways, with IL-1β playing a significant role.^637^ Normal endometrial stromal cells express VEGF at baseline, but its levels rise in response to estrogen and progesterone.^637,638^ Patients with endometriosis exhibit increased VEGF concentrations in peritoneal fluid and ectopic lesions, fostering a pro-angiogenic environment.^639,640^ VEGFR1/VEGF signaling in macrophages and fibroblasts further promotes lesion growth and lymphangiogenesis.^641,642^

Hypoxia plays a crucial role in endometriosis pathogenesis. HIF-1α levels are significantly elevated in ovarian endometriomas, correlating with increased VEGF mRNA expression under hypoxic conditions.^643–645^ Additionally, VEGF-C and VEGF-D contribute to lymphangiogenesis in endometriotic lesions, as observed in patients undergoing laparoscopic surgery.^646^ VEGF-C levels are consistently elevated, with recent findings linking COUP-TFII deficiency in ectopic stromal cells to excessive VEGF-C production. This imbalance enhances immune cell migration and lymphatic vessel formation, driving disease progression.^647–649^

Elevated VEGF-A, VEGFR2, and MMP9 levels correlate with lesion development in animal models.^650^ Anti-angiogenic therapies such as pazopanib and sorafenib show potential in treating endometriosis. Pazopanib reduces lesion size by at least 45%, while sorafenib more effectively modulates VEGF levels, suggesting distinct therapeutic roles.^651^ Given the cyclical regeneration of the endometrium, angiogenesis should remain precisely regulated. Further research is essential to refine targeted interventions while preserving normal reproductive function.

## Therapeutic targeting of VEGF/VEGFR signaling

### Current anti-VEGF/VEGFR therapies

The development of VEGF-targeting therapies has transformed treatment strategies for angiogenesis-driven diseases. Bevacizumab, a humanized anti-VEGF-A Ab, the first FDA-approved VEGF inhibitor (Table 2), marked a breakthrough in VEGF/VEGFR research.^652^ It has shown improved survival in metastatic colorectal cancer and other malignancies.^652,653^ Additionally, decoy receptors such as aflibercept sequester VEGF family members have further enhanced therapeutic efficacy.^654,655^

**Table 2:** FDA-approved drugs targeting VEGF/VEGFR signaling

Generic name	Brand name	Targets	Indications (approval year)	Mechanism of action (MOA)	Reference
				Mechanism of resistance	
Monoclonal antibody					
Bevacizumab	Avastin	VEGF-A	Colorectal cancer (2004), non-small cell lung cancer (2006), metastatic breast cancer (2008), glioblastoma (2009), renal cell carcinoma (2009), cervical cancer (2014), ovarian cancer (2018)	Monoclonal antibody that binds to VEGF-A, preventing it from activating VEGF receptors on the surface of endothelial cells	^709,710^
				Activation of autocrine VEGF signaling and hypoxia tolerance. Activation of other angiogenic pathways, such as FGF and PDGF, enhancement pericyte recruitment around blood vessels. Activation of autophagy and suppression of the Akt/mTOR pathway	
Bevacizumab-awwb	Mvasi	VEGF-A	Metastatic colorectal cancer, non-small cell lung cancer, glioblastoma, renal cell carcinoma, cervical cancer (2017)	A biosimilar to bevacizumab, it shares the same mechanism of action. Inhibiting the growth of blood vessels that supply tumors, essentially starving the tumor and slowing its growth	^711^
Ramucirumab	Cyramza	VEGFR2	Stomach cancer (2014), Gastric cancer (2014), non-small cell lung cancer (2014), colorectal cancer (2015), hepatocellular carcinoma (2019), metastatic EGFR-mutated non-small cell lung cancer (2020)	IgG1 monoclonal antibody that binds to VEGFR2, blocking VEGF ligands (VEGF-A/C/D) from activating the receptor, thereby inhibiting angiogenesis	^712–714^
				Resistance through alternative angiogenic pathways	
Ranibizumab	Lucentis	VEGF-A	Age-related macular degeneration (2006), retinal vein occlusion (2010), diabetic macular edema (2012), diabetic retinopathy (2015), myopic choroidal neovascularization (2017)	IgG1 antibody fragment specifically designed for ophthalmic use that binds to VEGF-A, blocking its activity in the eye	^715^
				Not well established, possible compensatory mechanisms in the eye	
Faricimab	Vabysmo	VEGF-A, Ang2	Age-related macular degeneration (2022), diabetic macular edema (2022), retinal vein occlusion (2023)	A bi-specific antibody targeting both VEGF-A and Ang2. By inhibiting VEGF-A, it reduces abnormal blood vessel formation and leakage, while inhibition of Ang-2 stabilizes blood vessels and reduces inflammation	^716^
				Resistance related to compensatory pathways and angiogenesis factors	
Recombinant fusion protein					
Aflibercept	Eylea	VEGF-A, VEGF-B, PIGF	Age-related macular degeneration (2011), diabetic macular edema (2014), retinal vein occlusion (2014), diabetic retinopathy (2019)	Trapping VEGF by binding VEGF-A, VEGF-B, and PIGF, thus preventing them from activating VEGF receptors	^717^
				Upregulation of alternative angiogenic factors, allow tumors or disease to bypass VEGF inhibition	
ziv-Aflibercept	Zaltrap	VEGF-A, VEGF-B, PIGF	Metastatic colorectal cancer (2012)	Trapping VEGF by binding VEGF-A, VEGF-B, and PIGF, thus preventing them from activating VEGF receptors	^718^
Oligonucleotide aptamer					
Pegaptanib	Macugen	VEGF-A165 isoform	Age-related macular degeneration (2004)	RNA aptamer that binds to and inhibits VEGF	^719^
Tyrosine kinase inhibitor					
Pazopanib	Votrient	VEGFR1-3, KIT, PDGFRα/β	Renal cell carcinoma (2009), soft tissue sarcoma (2012)	Oral tyrosine kinase inhibitor that targets multiple growth factor receptors, including VEGFRs, reducing angiogenesis and tumor growth	^720,721^
				Resistance because of mutations or activation of compensatory pathways	
Sorafenib	Nexavar	VEGFR1-3, KIT, RAF, PDGFRβ, FLT3, RET	Renal cell carcinoma (2005), Hepatocellular carcinoma (2007), differentiated thyroid carcinoma (2013)	Oral multi-kinase inhibitor that blocks various receptors involved in tumor growth and angiogenesis	^722^
				Resistance via secondary mutations in target receptors or alternative angiogenic pathways	
Sunitinib	Sutent	VEGFR1-3, KIT, CSF1R, PDGFRα/β, FLT3, RET	Renal cell carcinoma (2006), gastrointestinal stromal tumors (2006), pancreatic neuroendocrine tumors (2011)	Oral tyrosine kinase inhibitor targeting multiple pathways involved in angiogenesis and tumor growth	^723–726^
				Revival of angiogenesis through the activation of VEGF-independent pathways including PTEN downregulation and AKT/mTOR inhibition, a reduced bioavailability either through increased efflux or lysosomal sequestration	
Axitinib	Inlyta	VEGFR1-3, KIT, PDGFRα/β	Advanced renal cell carcinoma (2012)	A small molecule and orally bioavailable inhibitor of the ATP-binding domains of VEGFR1,2,3 of tyrosine kinases, axitinib reduces angiogenesis, leading to slower tumor growth and potentially shrinking tumors	^727,728^
				LINC00467, one of the lncRNAs found in cancers, induces axitinib resistance of HCC through miR-509-3p/PDGFRA axis	
Cabozantinib	Cabometyx,	VEGFR2, MET, AXL, RET, FLT3, TIE2, RON, KIT	Advanced renal cell carcinoma (2016), hepatocellular carcinoma (2019), differentiated thyroid cancer (2021)	Inhibits angiogenesis and tumor cell growth by blocking multiple pathways that support tumor survival and progression	^729–731^
				Resistance arises from the activation of alternative signaling pathways, including FGF and HGF, circulating immune cells induce resistance via increased secretion of pro-angiogenic factors	
	Cometriq		Metastatic medullary thyroid cancer (2012)	A biosimilar to cabometyx, it shares the same mechanism of action	
Lenvatinib	Lenvima	VEGFR1-3, KIT, RET, PDGFRα, FGFR1-4	Differentiated thyroid cancer (2015), advanced renal cell carcinoma (2016), hepatocellular carcinoma (2018), endometrial carcinoma (2019)	Blocks various growth factor receptors to inhibit angiogenesis and cancer growth	^732,733^
				Resistance develops through upregulation of compensatory angiogenic factors or activation of escape pathways by EMT, DNA damage, ferroptosis, autophagy, lncRNA, RNA modification, and cytokines	
Regorafenib	Stivarga	VEGFR1-3, KIT, TIE2, PDGFRβ, FGFR1, RET	Advanced colorectal cancer (2012), advanced gastrointestinal stromal tumor (2013), hepatocellular carcinoma (2017)	A small molecule inhibitor of multi-kinase that reduces angiogenesis and tumor cell survival through broad-spectrum kinase inhibition	^734–736^
				Regorafenib induces early senescence-like state as a cell death escape mechanism or activation of alternative pathways such as PI3K/AKT	
Vandetanib	Caprelsa	VEGFR2, EGFR, RET	Advanced medullary thyroid cancer (2011)	An orally active, small molecule, multi-targeted tyrosine kinase inhibitor blocks binding to VEGFR2/3, EGFR, RET tyrosine kinase receptor inducing angiogenesis and cell proliferation, slowing cancer growth	^737–739^
				EGFR mutations, secondary RET mutations, or upregulation of MET	
Ponatinib	Iclusig	VEGFR, PDGFR, EPH, FGFR, BCR-ABL1, SRC, RET, KIT	Leukemia (2012), resistant or intolerant chronic-phase chronic myeloid leukemia (2020), Philadelphia chromosome+ acute lymphoblastic leukemia (2024)	Tyrosine kinase inhibitor that targets BCR-ABL1 including a tough-to-treat mutant form (T315I) resisting many other drugs. In addition to BCR-ABL1, by inhibiting VEGFR, FGFR, PDGFR, KIT, and FLT3, it can slow down cancer growth, reduce blood supply to the tumor and help kill cancer cells	^740,741^
				Resistance occurs through additional mutations in BCR-ABL1 or activation of alternative pathways such as PI3K/AKT	
Apatinib	Aitan	VEGFR2, PDGFRβ, SRC, KIT, RET	Advanced gastric cancer (2014)	Small-molecule tyrosine kinase inhibitor that primarily targets VEGFR2. It blocks the signaling of VEGFR2, effectively reduces the supply of oxygen and nutrient to the cancer cells by limiting the tumor’s ability to form new blood vessels	^742–744^
				Upregulation of DUSP1 as a regulator of MAPK and increase of asparagine and glutamate in amino acid response pathway	
Nintedanib	Ofev	VEGFR1-3, PDGFRα/β, FGFR1-3, FLT3	Idiopathic pulmonary fibrosis (2014), systemic sclerosis or scleroderma (2019), chronic fibrosing interstitial lung diseases (2020)	Oral tyrosine kinase inhibitor that works by blocking tyrosine kinases involved in angiogenesis, fibrosis, and inflammation.	^745,746^
				Resistance through ATP-binding-cassette transporter B1 (ABCB1) overexpression	

Recent clinical trials continue to highlight the importance of anti-VEGF therapies. For instance, the PAOLA-1 trial (NCT02477644) demonstrated that combining olaparib, a PARP (poly ADP-ribose polymerase) inhibitor targeting DNA damage repair, with bevacizumab provides a significant survival benefit in ovarian cancer patients with homologous recombination deficiency-positive tumors.^656^ Similarly, the BEACON CRC trial (NCT02928224) showed that the combination of encorafenib (a BRAF inhibitor specifically targeting the BRAF V600E mutation), cetuximab (an anti-EGFR antibody), and bevacizumab substantially improves overall survival in BRAF-mutated metastatic colorectal cancer.^657^ Likewise, the RELAY study (NCT02411448) demonstrated that ramucirumab (anti-VEGFR2 Ab) plus erlotinib (EGFR TKI) significantly improved progression-free survival in EGFR-mutated metastatic non-small cell lung cancer (NSCLC), regardless of baseline mutations or resistance-associated alterations.^658^ Liquid biopsy revealed worse outcomes in patients with detectable circulating EGFR mutations, but VEGFR2 inhibition still provided benefits, supporting its role in overcoming EGFR-TKI resistance. The VEGFR inhibition has also demonstrated strong clinical benefits, particularly in HCC. The REACH-2 trial (NCT02435433) validated that ramucirumab provides significant survival benefits in patients with high levels of alpha-fetoprotein, a biomarker commonly used in HCC.^659^ Furthermore, combining VEGF inhibition with immune checkpoint and epigenetic regulators has emerged as a promising strategy to enhance VEGF-targeted therapy, potentially offering synergistic benefits.^660^ The CAPability-01 trial (NCT04724239) showed that addition of bevacizumab to sintilimab (anti-PD-1 Ab) and chidamide (histone deacetylase inhibitor) significantly improved survival and response rates in microsatellite (MSS)/proficient mismatch repair (pMMR) colorectal cancer, further supporting the rationale for combinatorial treatment strategies.

In ophthalmology, novel anti-VEGF approaches are redefining treatment paradigms. Smaller molecules designed for rapid ocular penetration have revolutionized AMD management. Ranibizumab, a humanized anti-VEGF-A Ab fragment, and brolucizumab, a single-chain anti-VEGF-A Ab, continue to improve retinal disease outcomes.^661,662^ The NORSE-EIGHT clinical trial validated ranibizumab’s superior efficacy over bevacizumab in improving best-corrected visual acuity.^663^ Notably, bevacizumab, under the brand name LYTENAVA™, has received regulatory approval for wet AMD treatment in the EU and UK.^664^

Collectively, these advancements in anti-VEGF/VEGFR therapies reinforce their integral role across oncology and ophthalmology. As combination approaches and biomarker-driven treatments continue to emerge, optimizing dosing strategies and overcoming resistance remain key priorities in the field.

### Biomarkers for VEGF/VEGFR pathway activity

Identifying biomarkers for VEGF/VEGFR activity is critical for optimizing therapy and predicting patient outcomes. Circulating VEGF levels, sVEGFRs, and Ang2 are commonly studied biomarkers. Elevated VEGF levels correlate with poor prognosis in cancers such as breast and lungs,^665^ whereas sVEGFR1 has been linked to pre-eclampsia and various cancers. Ang2 complements VEGF as a marker of vascular instability and disease severity.^666^ Additionally, epigenetic markers such as DNA methylation and circulating miRNAs provide minimally invasive tools for monitoring pathway activity.^667,668^

Advanced imaging techniques, including VEGF-specific PET tracers, enable real-time assessment of VEGF pathway activity, enhancing treatment precision.^669^ Additionally, transcriptomic and proteomic analyses have identified unique biomarker signatures, establishing a foundation for personalized therapy. Ongoing research continues to explore novel biomarkers, aiming to refine therapeutic strategies and improve patient outcomes. Furthermore, microvessel density (MVD) in tumor tissues indirectly reflects VEGF activity and correlates with poor outcomes in cancers such as breast and colorectal cancer.^670,671^ Circulating endothelial progenitor cells (EPCs) also serve as indicators of vascular repair and VEGF activity, with reduced EPC levels linked to impaired angiogenesis in cardiovascular diseases.^672–674^

### Emerging paradigms in anti-VEGF/VEGFR therapies and innovative strategies

The multifaceted biology of VEGF presents challenges for anti-VEGF/VEGFR therapies. Several mechanisms contribute to their enhanced efficacy: (1) anti-angiogenesis, targeting immature and leaky tumor vasculature to inhibit growth.^7^ (2) sensitization to chemotherapy, enhancing the anti-EC effects of low-dose chemotherapy by blocking EC survival.^675^ (3) vessel normalization, improving blood flow and enhancing delivery of therapeutics.^351^ (4) direct anti-tumor effects, interfering with tumor cell survival.^676^ (5) off-tumor benefits, improving cancer-associated systemic syndromes.^677,678^ (6) immune modulation, countering tumor immune privilege by enhancing dendritic cell (DC) differentiation.^679,680^ (7) reduced toxicity, mitigating chemotherapy-induced adverse effects such as bone marrow suppression.^681^

Resistance arises from alternative angiogenic pathways (e.g., FGF2, PDGF), vessel co-option, vasculogenic mimicry, or recruitment of myeloid-derived suppressor cells (MDSCs).^682–684^ Moreover, tumor heterogeneity and hypoxic/inflammatory microenvironments select resistant clones, necessitating novel approaches. To tackle resistance, combination therapies are actively explored. One strategy pairs anti-VEGF agent with FGF or Ang2 inhibitors. Another integrates anti-VEGF therapies with immune checkpoint inhibitors (ICIs), such as anti-PD-1/PD-L1 therapies, where tumor vessel normalization enhances immune cell infiltration.^351^ Consequently, FDA-approved regimens include atezolizumab (anti-PD-L1 Ab) plus bevacizumab for HCC,^685^ pembrolizumab (anti-PD-1 Ab) plus axitinib (TKI) for renal cell carcinoma,^686^ and atezolizumab (anti-PD-L1 Ab) plus bevacizumab (anti-VEGF-A Ab) with chemotherapy for NSCLC.^687^ Yet, efforts persist to optimize patient selection and minimize toxicity.^688,689^

**Fig. 10:**
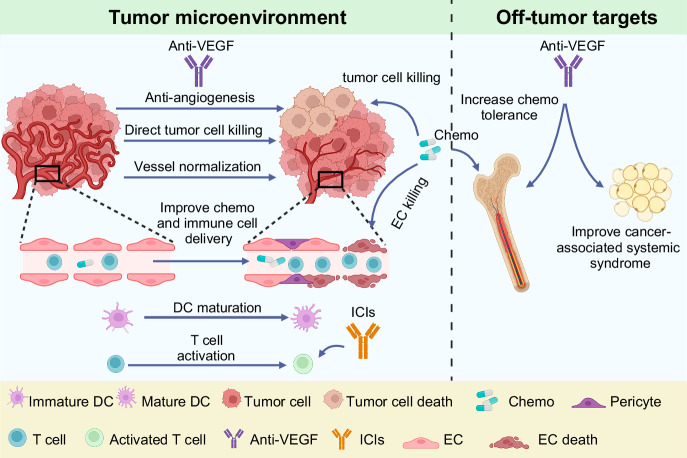
Mechanisms of action of anti-VEGF/VEGFR therapies in combination therapy. In the tumor microenvironment (TME), the mechanisms of action of anti-VEGF/VEGFR therapies combined with chemotherapy or immune checkpoint inhibitors (ICIs) include reducing angiogenicity of vessels for tumor inhibition, inducing vessel normalization for better chemo-drug or immune cell delivery, directly inhibiting tumor cell proliferation, increasing anti-endothelial cell effect of chemotherapy, and modulating immune cells for better dendritic cell (DC) differentiation and T-cell activation. Outside the TME, the anti-VEGF/VEGFR therapies may reduce chemotoxicity and improve cancer-associated systemic syndromes, thereby improving therapeutic efficacy. Created in BioRendender.com

Importantly, the theoretical framework for combining anti-VEGF/VEGFR therapies with ICIs involves two key mechanisms: (1) immune modulation, where VEGF inhibition reduces immunosuppression and transforms tumors into a “hot” phenotype more responsive to ICIs,^690,691^ and (2) vessel normalization, which enhances immune infiltration by improving vascular perfusion.^692^ Yet, critical issues remain. Considering the anti-tumor effects observed in immunodeficient models, it remains unclear to what degree VEGF/VEGFR inhibition relies on immunomodulation. Additionally, in “non-angiogenic” tumors resistant to these therapies,^693^ can the immunomodulatory benefits of combination therapy provide an advantage? While vessel normalization improves immune infiltration, excessive vascular reduction may impair it, underscoring the need to balance normalization with maintaining adequate vascular density. Moreover, unraveling the interplay between TME dynamics and VEGF signaling could unlock new strategies to overcome these limitations (Fig. 10).

To address these challenges, proteolysis-targeting chimeras (PROTACs) have emerged as a novel modality. Additionally, antibody-drug conjugates (ADC) targeting VEGF-VEGFR pathways represent another promising modality, combining precise targeting of angiogenic signaling with the delivery of cytotoxic agents to inhibit tumor growth and angiogenesis.^694^ Concurrently, artificial intelligence (AI) is revolutionizing drug discovery by identifying dual-target VEGFR inhibitors with greater precision.^424^ Coupled with genomic and transcriptomic profiling, AI provides critical insights into oncogenic pathways, enabling biomarker development and rational combination therapies. Despite progress, toxicity and biomarker gaps hinder adoption, necessitating refined patient selection. These advances signal a shift toward personalized, multi-modal approaches, tackling resistance and expanding therapeutic horizons.

## Conclusion and perspective

The VEGF/VEGFR signaling pathway remains central to angiogenesis, lymphangiogenesis, and vascular remodeling, affecting diverse physiological and pathological processes. Over the past few decades, the development of VEGF-targeted therapies has transformed the treatment landscape for cancer, ocular diseases, and inflammatory disorders. Monoclonal antibodies, tyrosine kinase inhibitors, and VEGF-trap fusion proteins have significantly improved patient survival and quality of life. However, therapeutic resistance, systemic toxicities, and limitations in predictive biomarkers continue to present challenges.

One of the major challenges in anti-VEGF therapy is the emergence of compensatory pro-angiogenic pathways. The upregulation of FGF2, Ang, and the induction of vasculogenic mimicry - where tumor cells adopt endothelial-like characteristics - contribute to tumor escape from VEGF inhibition. Similarly, VEGF-C and VEGF-D-mediated lymphangiogenesis facilitates cancer progression and metastasis, emphasizing the need for combinatorial targeting strategies. In wet AMD and DR, VEGF-C/D upregulation has been implicated in resistance to anti-VEGF-A monotherapies. The ongoing development of VEGF-C/D inhibitors, such as OPT-302,^529^ represents a promising approach to overcoming this resistance.

The role of VEGFs extends beyond angiogenesis, impacting fibrosis, metabolic syndromes, and neurodegeneration. VEGF-B, traditionally considered minimally angiogenic, has emerged as a critical player in neuroprotection, with potential implications for neurodegenerative disorders such as Alzheimer’s disease.^695,696^ In addition, VEGF signaling contributes to endothelial dysfunction in diabetes and cardiovascular disease, highlighting its potential as a therapeutic target in metabolic disorders. Moreover, recent findings show VEGF-B inhibits angiogenesis by suppressing FGF2/FGFR1 signaling and forming VEGFR1/FGFR1 complexes that block ERK activation.^137^ This raises concerns about therapies including aflibercept, which targets both VEGF-A and VEGF-B, as blocking VEGF-B may disrupt its regulatory function in pathological angiogenesis.

Advancements in precision medicine have introduced new opportunities for optimizing VEGF-targeted therapies. AI-driven biomarker identification and spatial transcriptomics hold promises for improving patient stratification and treatment personalization. However, the standardization of predictive biomarkers remains a challenge due to tumor heterogeneity and the dynamic nature of VEGF signaling. For instance, VEGF-A+ fibroblasts have been proposed as a biomarker in HCC,^697^ yet their clinical validation across diverse patient cohorts is still lacking. Future efforts should focus on refining biomarker discovery approaches to enhance their clinical utility. Additionally, identifying non-invasive biomarkers, such as circulating exosomal VEGF or imaging-based vascular signatures, could improve real-time monitoring of treatment responses.

To address systemic toxicities associated with VEGF inhibition, emerging drug modalities offer alternative solutions. Long-acting gene therapy, nanoparticle-encapsulated siRNA, and CRISPR-based VEGF modulation represent promising strategies to mitigate hypertension, proteinuria, and impaired wound healing. Additionally, newer modalities such as bi-specific antibodies (e.g., faricimab, targeting both VEGF and Ang2) and VEGF-trap fusion proteins (e.g., conbercept in ocular diseases) provide enhanced specificity and efficacy. Recent advances in ADCs and PROTACs have further expanded the landscape of VEGF-related interventions, enabling more selective and potent therapeutic strategies. These approaches warrant further clinical exploration to determine their safety, efficacy, and long-term benefits.

Furthermore, research into the interplay between VEGF and other angiogenic factors is essential for refining combination therapies. Dual inhibition of VEGF and HIF-1α has shown promise in overcoming hypoxia-induced resistance, while VEGF and PDGFRβ co-targeting strategies aim to enhance vascular normalization and drug delivery in tumors.^698,699^ Additionally, integrating VEGF-targeted approaches with ICIs continues to gain momentum, particularly in cancers with immunosuppressive microenvironments. A deeper mechanistic understanding of these interactions will be critical for optimizing treatment regimens and improving patient outcomes.

Future research should also explore the impact of VEGF inhibition in aging and regenerative medicine. As the aging population grows, understanding the consequences of long-term VEGF suppression on vascular integrity, wound healing, and neurovascular health is crucial. VEGF plays a pivotal role in tissue repair and homeostasis, and prolonged inhibition may contribute to frailty, cognitive decline, and impaired organ regeneration. Balancing VEGF suppression for disease treatment while preserving its physiological functions will be essential for minimizing unintended side effects.

Additionally, continued investigation into the role of VEGFs in inflammatory and autoimmune diseases is warranted. VEGF has been implicated in conditions such as RA, psoriasis, and multiple sclerosis, where its modulation could provide therapeutic benefits. Exploring VEGF-targeting strategies in these diseases could expand the clinical applications of anti-angiogenic therapies beyond oncology and ophthalmology. Furthermore, understanding the interplay between VEGF signaling and the gut microbiome may offer novel insights into angiogenesis-related metabolic disorders, paving the way for innovative treatment approaches.

In conclusion, VEGF-targeted therapies have fundamentally reshaped disease management. However, their future success depends on overcoming resistance, enhancing treatment precision, and exploring novel applications beyond angiogenesis. Continued investigation into VEGF signaling complexity, combined with cutting-edge technological innovations, will be essential in maintaining VEGF inhibitors as a cornerstone of therapeutic intervention. As these efforts progress, VEGF-targeted therapies will continue to hold transformative potential across cancer, ocular diseases, and other fields, offering new hope to patients.
